# Nucleolar DEAD-Box RNA Helicase TOGR1 Regulates Thermotolerant Growth as a Pre-rRNA Chaperone in Rice

**DOI:** 10.1371/journal.pgen.1005844

**Published:** 2016-02-05

**Authors:** Dong Wang, Baoxiang Qin, Xiang Li, Ding Tang, Yu’e Zhang, Zhukuan Cheng, Yongbiao Xue

**Affiliations:** 1 State Key Laboratory of Molecular Developmental Biology, Institute of Genetics and Developmental Biology, Chinese Academy of Sciences and National Center for Plant Gene Research, Beijing, China; 2 State Key Laboratory of Plant Genomics, Institute of Genetics and Developmental Biology, Chinese Academy of Sciences and National Center for Plant Gene Research, Beijing, China; 3 Collaborative Innovation Center for Genetics and Development, Fudan University, Shanghai, China; 4 Beijing Institute of Genomics, Chinese Academy of Sciences, Beijing, China; Shanghai Institute of Plant Physiology and Ecology, Shanghai Institutes for Biological Sciences (SIBS), CHINA

## Abstract

Plants have evolved a considerable number of intrinsic tolerance strategies to acclimate to ambient temperature increase. However, their molecular mechanisms remain largely obscure. Here we report a DEAD-box RNA helicase, TOGR1 (Thermotolerant Growth Required1), prerequisite for rice growth themotolerance. Regulated by both temperature and the circadian clock, its expression is tightly coupled to daily temperature fluctuations and its helicase activities directly promoted by temperature increase. Located in the nucleolus and associated with the small subunit (SSU) pre-rRNA processome, TOGR1 maintains a normal rRNA homeostasis at high temperature. Natural variation in its transcript level is positively correlated with plant height and its overexpression significantly improves rice growth under hot conditions. Our findings reveal a novel molecular mechanism of RNA helicase as a key chaperone for rRNA homeostasis required for rice thermotolerant growth and provide a potential strategy to breed heat-tolerant crops by modulating the expression of *TOGR1* and its orthologs.

## Author Summary

Global warming is increasingly posing negative impacts on crop productivity. In this study, we report a nucleolar-located RNA helicase TOGR1 for thermotolerant growth in rice. TOGR1 maintains pre-rRNA homeostasis under high temperature by securing a proper pre-rRNA structure via elevating its helicase activity. Its expression is high temperature inducible with an afternoon peak expression, consistent with a high temperature anticipation of the circadian clock. Transcriptome analysis revealed that TOGR1 is essential in coordinating primary metabolisms to support thermotolerant growth. Importantly, an enhanced expression of TOGR1 significantly increased biomass of rice. Our findings reveal a novel role of a RNA helicase in thermotolerance and provide a potential strategy to breed heat-tolerant rice cultivars and possibly other heat-tolerant crops.

## Introduction

Temperature rising caused by global warming has imposed significant negative effects on crop yields and most likely the damage level will keep rising in future [1,2]. On the other hand, the sessile lifestyle of plants necessitates specific adaptations to geographical variations of environmental temperatures in their living regions as well as to extensive temperature fluctuations caused by the day-night cycle, weather variations and seasonal changes [3]. To survive in these often stressful conditions, they have evolved a number of intrinsic tolerance strategies to adapt to high temperatures. Heat shock protein protection, membrane lipid unsaturation and reactive oxygen species scavenging have been all reported to confer thermotolerance to plants [4–6]. It was also reported that alternative histone H2A.Z coordinates a high temperature transcriptome [7] and a transcription regulator ELF3 controls plant thermoresponsive growth [8].

Ribosomal RNA homeostasis is crucial to normal growth and development. It is well known that RNA homeostasis is affected by cold stress, and protected by RNA helicases [9], which mediate RNA conformational changes by hydrolyzing ATP and unwinding short RNA duplexes adjacent to their binding sites in a nonprocessive way [10,11]. They are widely involved in diverse cellular processes such as transcription, RNA splicing, RNA transport, degradation and translation, and have been well documented to be involved in cold stress responses in both bacteria and yeast [9,12]. So far, at least 7 yeast RNA helicases have been shown to associate with the SSU in the nucleolus [13,14], which is essential for pre-rRNA processing [15]. One of those SSU associated RNA helicases, Rrp3 has been found to support an effective pre-rRNA processing required for cell proliferation [14]. Higher plants possess a larger and more diverse family of RNA helicases than other organisms [16]. For example, at least 73 RNA helicases are encoded by the rice genome [17]. This large number of RNA helicases suggests a predominant role of them in modulating cellular response to a diverse range of abiotic stresses encountered by plants [18,19]. Among them, one DEAD-box RNA helicase LOS4 has been shown to modulate chilling resistance by facilitating mRNA export from the nucleus to the cytoplasm [20,21], and another member of this family AtRH25 also confers cold stress tolerance through an unknown mechanism [18], while several other members appear to function in salinity stress tolerance [22–24]. However, little is known about the potential role of RNA helicases at high temperatures.

Rice is a staple food crop initially domesticated in subtropical regions [25], where it must cope with environmental challenges caused by high temperatures, and subsequent human selection has extended its cultivation to temperate zones with enhanced chilling tolerance [26]. It has been recently reported that a proteasome α2 subunit gene [27] and a receptor-like kinase ERECTA [28] both contribute to rice thermotolerance. Another recently identified gene *COLD1* was reported to confer chilling tolerance in *japonica* rice by regulating G-protein signaling pathway [26]. However, the underlying molecular mechanisms of this temperature adaptation still remain mostly elusive. Here we report on TOGR1, a DEAD-box RNA helicase protecting rice growth at high temperatures as an intrinsic pre-rRNA chaperone. Consistent with this role, we also found that it is regulated by both temperature and the circadian clock. Furthermore, TOGR1 is recruited to the SSU in the nucleolus to facilitate an effective pre-rRNA processing required for normal cell division and thus plant growth at high temperature. Transcript level of this gene is positively correlated with plant height in different cultivated varieties and wild rice species, and its overexpression significantly improves rice growth at high temperatures. Our results uncover an essential molecular mechanism of RNA helicase as a key chaperone for rRNA biogenesis required for plant thermotolerant growth, and a possibility to produce heat-tolerant crops by regulating the expression of TOGR1 and its orthologs.

## Results

### Identification of a Thermosensitive Dwarf Rice Mutant

**Fig 1:**
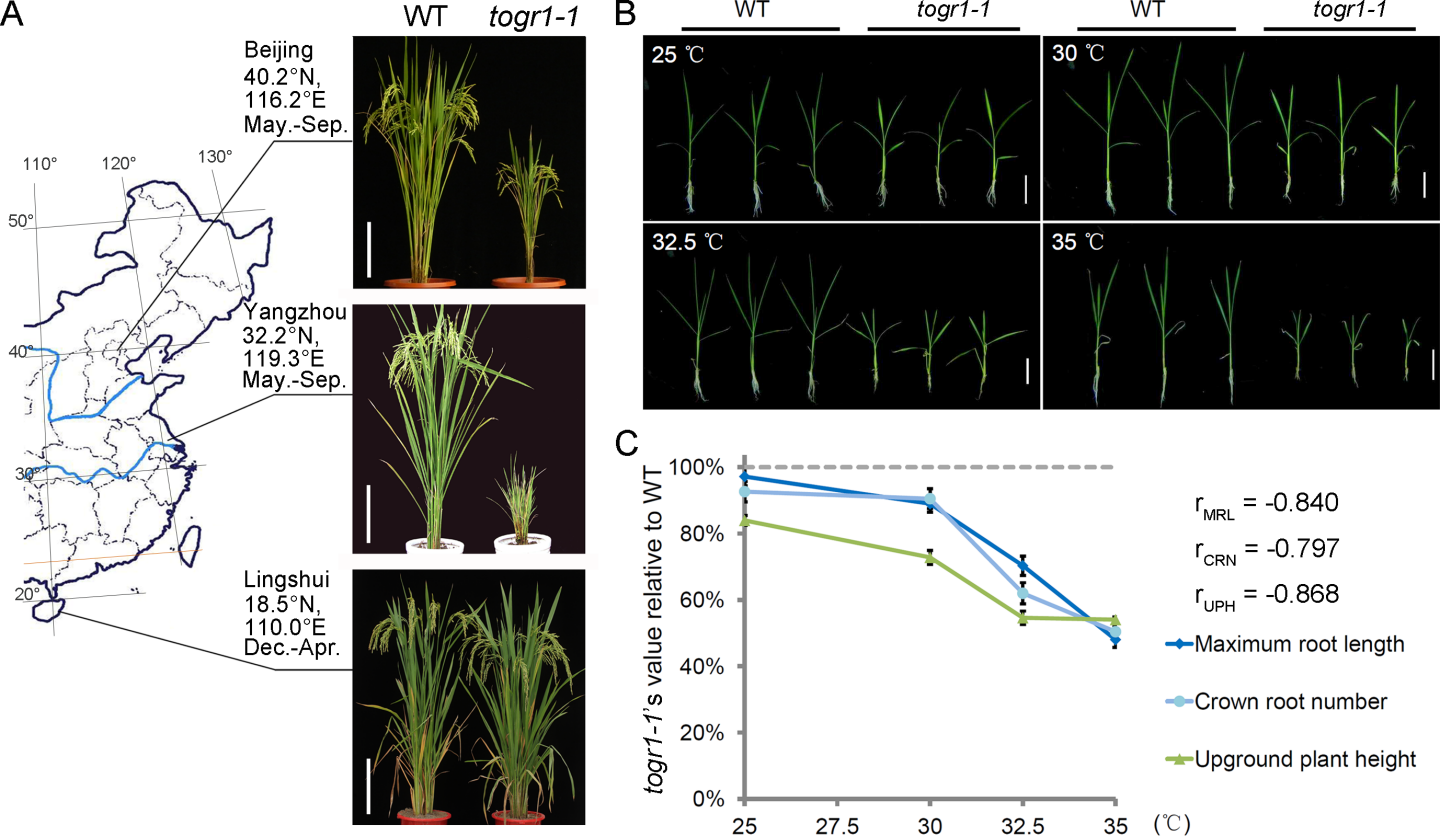
*togr1-1* is a thermosensitive dwarf mutant. **(A)** Wild-type (WT) Zhongxian 3037 and the thermosensitive dwarf mutant (*togr1-1*) grown in fields of three locations in China under different temperature conditions. Growing seasons are: Beijing and Yangzhou, summer-autumn; Lingshui, winter-spring. Scale bars: 20 cm. **(B and C)** Growth of WT and *togr1-1* seedlings at four different temperatures. Newly geminated seedlings were grown in climate chambers at indicated temperatures for three weeks before photographing and measurement. Sensitivity of the mutant to high temperature was evaluated by comparing maximum root length (MRL), crown root number (CRN) and upground plant height (UPH) between the WT and *togr1-1*. Data are represented as mean ± SEM (n = 15 plants). Scale bars: 5 cm. r_MRL_ = -0.840; r_CRN_ = -0.797; r_UPH_ = -0.868; p<0.001; Pearson product-moment correlation coefficient.

To understand the intrinsic molecular mechanisms of rice acclimating to at high temperatures, we took advantage of a wide range of climate variations in China’s rice growing regions and carried out a genetic screen for temperature-sensitive mutants from a collection of rice varieties. Among them, a spontaneous recessive thermosensitive dwarf mutant (*thermotolerant growth required1-1*, *togr1-1*) was isolated from an *indica* variety, Zhongxian 3037. When planted in paddy fields at three locations representing three divergent temperature regimes (S1 Fig), *togr1-1* exhibited a dramatic growth phenotypic variation (Fig 1A; S2A–S2E Fig). Grown in Yangzhou’s hot summer-autumn with a highest daily maximum temperature near 38°C, 23 days exceeding 34°C, which is considered to be a heat-stress threshold temperature for rice [29], and 71 days exceeding 30°C, *togr1-1* was extremely dwarf with narrow leaf blades and did not set any seed. Similarly, under Beijing’s summer-autumn conditions (daily maximum temperatures up to 36°C, 15 days exceeding 34°C and 72 days exceeding 30°C), *togr1-1* was dwarf with narrow leaf blades before September, then recovered to semi-dwarf when temperature was cooling down, and finally produced small panicles with a few seeds. By contrast, in the cool winter-spring of Linshui (daily maximum temperatures during vegetative growth stage generally below 28°C and never reaching 30°C), *togr1-1* only showed a slight overall growth difference from wild-type. To further confirm the temperature dependence of the phenotypic variations of *togr1-1*, we tested its effect on growth of the mutant seedlings under controlled conditions. After grown in a climate chamber at different temperatures for three weeks, upground plant height, maximum root length and crown root number were determined for WT and *togr1-1* seedlings (Fig 1B and 1C; S2F–S2H Fig). The mutant seedlings showed significantly reduced plant height, root length and crown root number at 30°C, 32.5°C and 35°C, whereas they grew similarly to wild-type at 25°C except for a slightly reduced plant height. Furthermore, the relative values of all of the three examined variables of the mutant seedlings compared to the wild-types showed significant strong negative correlations with temperature (Fig 1C), supporting that the retarded growth of *togr1-1* is high temperature dependent. To examine the morphological and cellular basis of the *togr1-1* dwarfism, the plant architectures of *togr1-1* grown in Beijing’s hot summer were examined at tillering stage. Its leaf blades were narrowed mainly due to a decrease in vascular bundle and lateral vein numbers, and all internodes were shortened (S3A and S3B Fig). Furthermore, cross-sections of leaf sheath, leaf blades, internodes and root maturation zones revealed that *togr1-1* and wild-type did not show a significant difference in cell elongation but a reduction in cell numbers (S3C–S3E Fig), indicating that *TOGR1* is required for normal cell division at high temperatures.

### *TOGR1* Encodes a Temperature-Dependent and Circadian-Regulated DEAD-Box RNA Helicase

**Fig 2:**
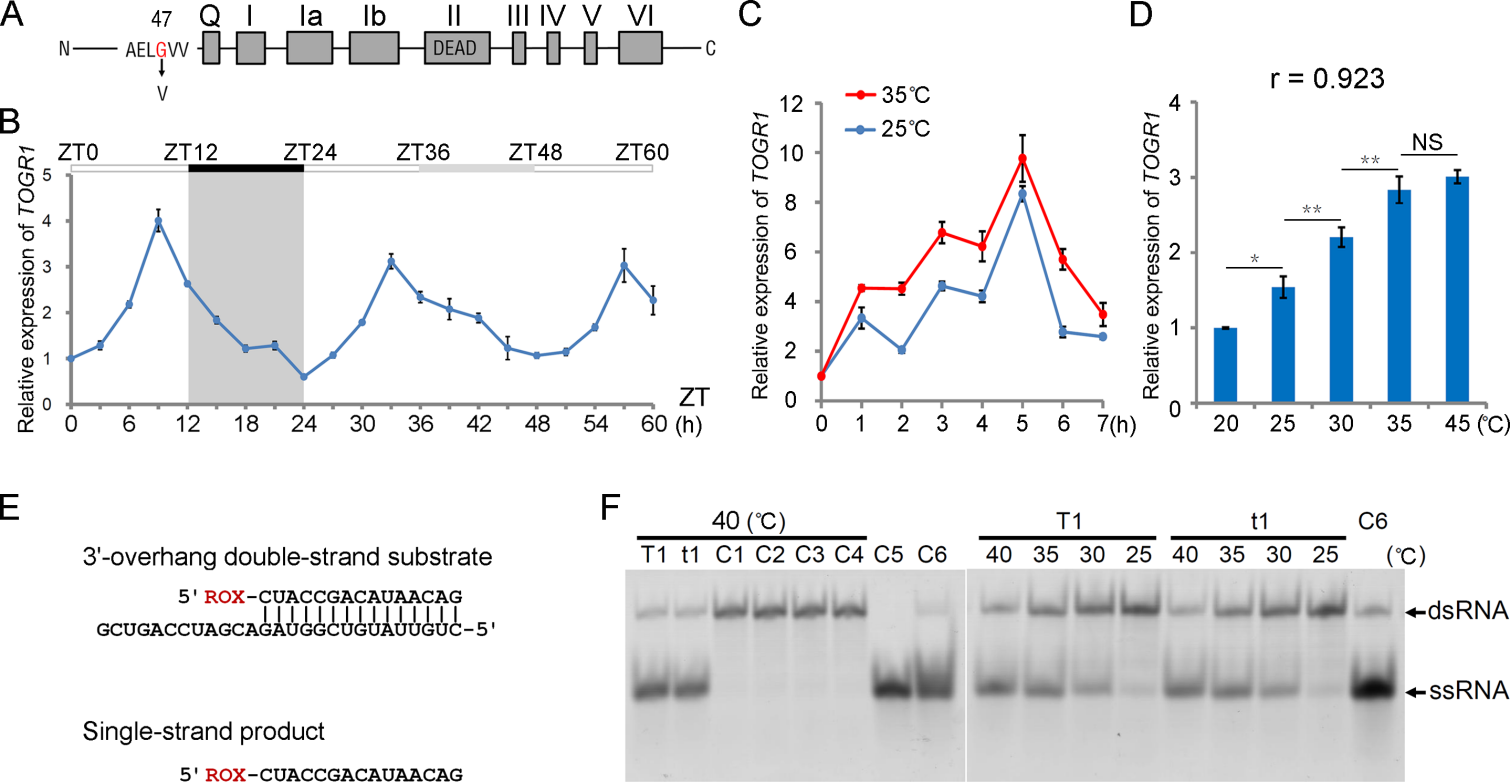
*TOGR1* encodes a DEAD-box RNA helicase under control of both temperature and the circadian clock. **(A**) Schematic diagram shows the TOGR1 protein containing nine canonical motifs of DEAD-box RNA helicases. The mutation site of togr1-1 (G to V) is indicated by an inverted vertical arrow. **(B)** Circadian expression pattern of *TOGR1*. Plants were entrained in 12-h-light/12-h-dark conditions and subsequently grown under constant light conditions (ZT0-ZT12, light; ZT12-ZT24, dark; ZT24-ZT60, constant light; temperature, 30°C). RNA was extracted from leave blades and analyzed by qRT-PCR. Data are represented as mean ± SEM (n = 3 replicates). **(C)** Time course analysis of the effect of high temperature on transcription of *TOGR1*. Two-week-old WT Zhongxian3037 seedlings grown under 12-h-light-25°C/12-h-dark-20°C conditions were transferred to 35°C at ZT4. Transcript level of *TOGR1* was analyzed every one hour. Seedlings kept at 25°C were used as control. **(D)** Transcript level of *TOGR1* under different temperatures. **(C and D)** Total RNA was extracted from seedlings. Data are represented as mean ± SEM (n = 3 replicates). NS, not significant; *p < 0. 05; **p<0.01; one-way ANOVA, *a priori* contrasts. For correlation analysis, r = 0.923; p<0.001; Pearson product-moment correlation coefficient. **(E)** Schematic representations of the 5'-ROX labeled 3'-overhang RNA substrate and the single-strand RNA product detected in helicase assay after unwinding. **(F)** TOGR1 is capable of unwinding RNA duplexes. Helicase activity of TOGR1 (T1) and togr1-1 (t1) on the 3'-overhang RNA duplex were analyzed at indicated temperatures, respectively. Products were detected after 30 min incubation unless otherwise specified. C1, TOGR1 without ATP; C2, togr1-1 without ATP; C3, Precision protease; C4, 3'-overhang substrate; C5, 5'-ROX labeled single strand RNA; C6, 3'-overhang substrate boiled for 5 min. Sizes of the double strand RNA substrate (dsRNA) and the single strand product (ssRNA) are indicated.

To isolate the mutated gene that controls the thermosensitive phenotype of *togr1-1*, an F2 population for positional cloning was generated from a cross between *togr1-1* and a japonica variety Zhonghua-11, and was then grown in the paddy fields under Beijing’s hot summer conditions. We mapped it to a 28.5-kb region of chromosome 3 (S4A Fig), and found a single nucleotide G to T substitution at position 140 of the first exon of *LOC_Os03g46610* (named as *TOGR1*), leading to a Gly to Val substitution at position 47 of amino acid sequence (Fig 2A). When *togr1-1* was transformed by a construct containing an entire ORF of *TOGR1* and its putative promoter sequence, the transgenic plants exhibited normal growth under Beijing’s hot summer conditions (S4B and S4C Fig). Furthermore, three other allelic recessive mutants of *togr1* (*togr1-2* to *-4*) screened out from an EMS (ethyl methanesulfonate) induced mutation library using TILLING (targeting induced local lesions in genomes) method also showed a high temperature dependent growth repression (S5 and S6 Figs). This demonstrated that the *TOGR1* mutation is responsible for the thermosensitive phenotype.

To characterize the expression of *TOGR1*, GUS reporter fused to a putative promoter of *TOGR1* was used to transform plants and revealed that it is widely expressed in roots, shoots, leaves, culms, spikelets and anthers during different developing stages (S7 Fig), consistent with the microarray data from RiceXPro [30], indicating a general requirement of *TOGR1* by all organs. To examine if *TOGR1*’s expression is regulated by temperature, two-week-old Zhongxian3037 seedlings grown under 12-h-light-25°C/12-h-dark-20°C condition were transferred to 35°C at ZT4 and kept for seven hours. Transcription level of *TOGR1* after 0–7 h high temperature treatment was analyzed by qRT-PCR (Fig 2C). In comparison with its expression in the seedlings kept at 25°C, the highest level of transcription enhancement (two-fold) was detected after two hours high temperature treatment. Transcript level of *TOGR1* were then analyzed at 20, 25, 30, 35 and 40°C (Fig 2D) after two hours temperature treatment starting from ZT4, and exhibited a strong positive correlation with temperature, indicating that its expression is high temperature inducible. In Fig 2C, *TOGR1* reached peak expression after five hours treatment (ZT9) at both temperatures and the induction of high temperature at that time was not as significant as that after two hours treatment, indicating control of other mechanisms as well. To examine if the expression of *TOGR1* was controlled by the circadian clock, we kept temperature at constant 30°C and entrained two-month-old Zhongxian 3037 plants under 12-h-light/12-h-dark (L/D) condition, and subsequently detected its transcript level under both L/D and continuous light (L/L) conditions by qRT-PCR. The temporal expression level of *TOGR1* (Fig 2B) waved in a cosine pattern with a period that lasted approximately 24 h under both L/D and L/L conditions, indicating a circadian regulation of its expression. In addition, its peak expression appeared at zeitgeber time (ZT9) corresponding to afternoon of a subjective day, the hottest time of daily temperature fluctuation, indicating the circadian clock being used to anticipate high temperature in the afternoon and a coupled expression of *TOGR1* with a daily temperature alteration.

The amino acid sequence of TOGR1 contains nine motifs (Fig 2A; S8 Fig) which are widely conserved for ATP-dependent DEAD-box RNA helicases [31,32] and is highly similar to Rrp3 in *S*. *cerevisiae* [33] and DDX47 in *H*. *sapiens* [34] (S8 and S9 Figs), suggesting that TOGR1 is functionally similar to those pre-rRNA processing helicases.

To verify the RNA helicase activity of TOGR1 *in vitro* and examine any potential functional difference between TOGR1 and togr1-1, we expressed and purified both TOGR1 and togr1-1 using *E*. *coli* expression system (S10 Fig), and examined their activities on a randomly synthesized 3'-overhang RNA duplex (Fig 2E) at 25°C, 30°C, 35°C and 40°C, respectively. For both TOGR1 and togr1-1 (Fig 2F), after incubation with ATP for 30 min, the single strand RNA (ssRNA) corresponding to the product of unwinding the double strand RNA substrate (dsRNA) was accumulated and no apparent ssRNA products were detected for the experimental controls including TOGR1 and togr1-1 without ATP, and the Prescission protease control with ATP. This detected capability of unwinding RNA duplexes into ssRNA products in the presence of ATP indicates that both TOGR1 and togr1-1 have ATP-dependent RNA helicase activities *in vitro*. It is noteworthy that the unwinding activity of TOGR1 was markedly enhanced following a temperature increase from 25°C to 40°C (Fig 2F), indicating that temperature directly controls the helicase activities of TOGR1. Intriguingly, compared with TOGR1, neither the helicase activities nor the temperature sensitivity of togr1-1 was diminished, suggesting that this activity is not directly related to its defect (see below). Taken together, these results showed that TOGR1 encodes a temperature-dependent DEAD-box RNA helicase.

### TOGR1 Is Required for a Normal Pre-rRNA Processing Pathway at High Temperatures

**Fig 3:**
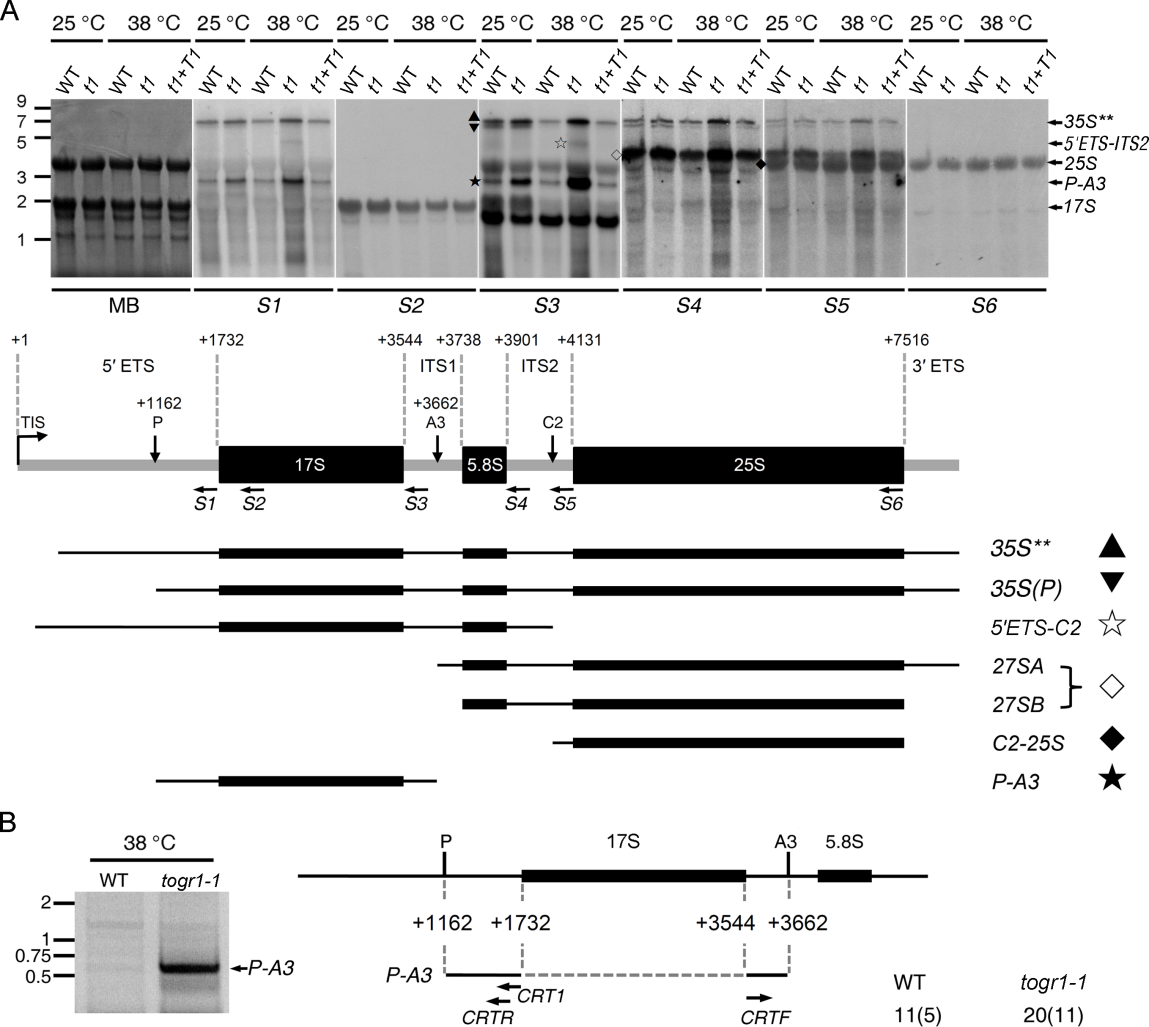
TOGR1 promotes an effective processing of rRNA intermediates at high temperatures. **(A)** Northern analysis of rRNA intermediates in WT and *togr1-1* plants. Total RNA extracted from WT and *togr1-1* leaves at 25°C and 38°C was separated on 1.2% denaturing agarose gel and hybridized with specific oligonucleotides (*S1*-*S6*) indicated by horizontal arrows in the schematic 35S precursor blow, in which the transcription initiation site (TIS, +1) and three of the endonucleolytic cleavage sites (P, +1162; A3, +3662; C2) are shown as well. Methylene blue staining of the blot (MB) and hybridizations with *S2* and *S6* were used as loading controls. Sizes of RNA markers (kb) are given on the left. Expected mature and intermediate rRNAs are labeled on the right. The detected rRNA intermediates are also designated with symbols together with diagrammatic representations (lower panel). **(B)** Detection of *P-A3* intermediate accumulated at 38°Cby circular RT-PCR. Negative image of EBr stained 1% agarose gel is shown. The indicated band was excised, cloned and sequenced. Sizes of DNA markers (kb) are indicated on the left. The amplified 5′ and 3′ extremities (solid lines) of *P-A3* relative to the 35S precursor are presented in the diagram. Oligonucleotide (horizontal arrow) *CRT1* was used to synthesize first strand cDNA and *CRTF* and *CRTR* were used in amplification. The number of sequenced clones and polyadenylated clones (in parentheses) for WT and *togr1-1* is given on the right.

The implication of yeast Rrp3 in pre-rRNA processing [14] prompted us to investigate the role of TOGR1 in the rRNA biogenesis pathway. we compared the pattern of pre-rRNA intermediate accumulation in *togr1-1* with that in wild-type as well as *togr1-1* and the complemented plants using seedlings first grown at 25°C for two weeks and then subjected to just one day of high temperature at 38°C. As controls, seedlings grown at continuous 25°C were also investigated. Total RNA isolated from these plants was analyzed by northern hybridizations using oligonucleotide probes *S1*-*S6* (Fig 3A; S1 Table). The most remarkable difference between wild-type and *togr1-1* was a 17S precursor *P-A3* detected by probe *S1* and *S3*, which consists of partial 5' ETS, mature 17S rRNA and partial ITS1 (Fig 3A). Under both temperature conditions, this intermediate was present at higher levels in *togr1-1* than in wild-type and the complemented plants, with the difference at 38°C more prominent. When *togr1-1* at 38°C and 25°C were further compared, a remarkable accumulation of *P-A3* was revealed for the higher temperature. By contrast, it was detected at a relatively low level in wild-type at 38°C in comparison with that at 25°C. Similar patterns were also detected for 35S**, the highest molecular weight intermediate identified by probes *S1*, *S3*, *S4* and *S5* and known as a precursor for 17S, 5.8S and 25S rRNA [35], and *27SA* plus *27SB* identified by probes *S4* and *S5* and known as precursors of 5.8S and 25S rRNA [35], although not as prominent as *P-A3*. Another apparent difference was that the intermediate *5'ETS-C2* detected by probes *S1*, *S3* and *S4* was only present in *togr1-1* at 38°C, but undetectable for wild-type and the complemented plants at both temperatures and for *togr1-1* at 25°C. This intermediate presumably spans regions 5' ETS, 17S rRNA, ITS1, 5.8S rRNA and partial ITS2. Taken together, these results indicated that in wild-type plants acclimating to heat, the *P-A3*, 35S** and *27SA&B* intermediates are normally reduced as a consequence of an elevated pre-rRNA processing rate upon a shift to high ambient temperatures and by contrast these intermediates plus *5'ETS-C2* were accumulated in *togr1-1* under the same conditions, indicating a significant delayed pre-rRNA processing in the *togr1-1* thermosensitive mutant.

To define the sequence of the *P-A3* intermediate, its 5' and 3' ends were further analyzed by circular RT-PCR using total RNA isolated from wild-type and *togr1-1* seedlings exposed to 38°C. In agreement with the northern blot analysis, it was revealed that the *P-A3* intermediate was released by cleavages at two sites corresponding to the P and A3 sites in *Arabidopsis* (Fig 3B; S11 Fig). The sequence of the A3 site (AAGGAAC) is conserved with that in *Arabidopsis*, providing a support to a previous hypothesis that this site is conserved within plants [36]. In addition, a significant higher level of the products was also obtained from *togr1-1* than wild-type as the same amount of RNA was used (Fig 3B).

Taken together, these results showed that TOGR1 is required to maintain a normal pre-rRNA processing pathway at high temperatures and the mutation of togr1-1 caused a delayed pre-rRNA processing.

### TOGR1 Is a Partner of the SSU Complex in the Nucleolus and Required for an Effective Cell Proliferation at High Temperatures

**Fig 4:**
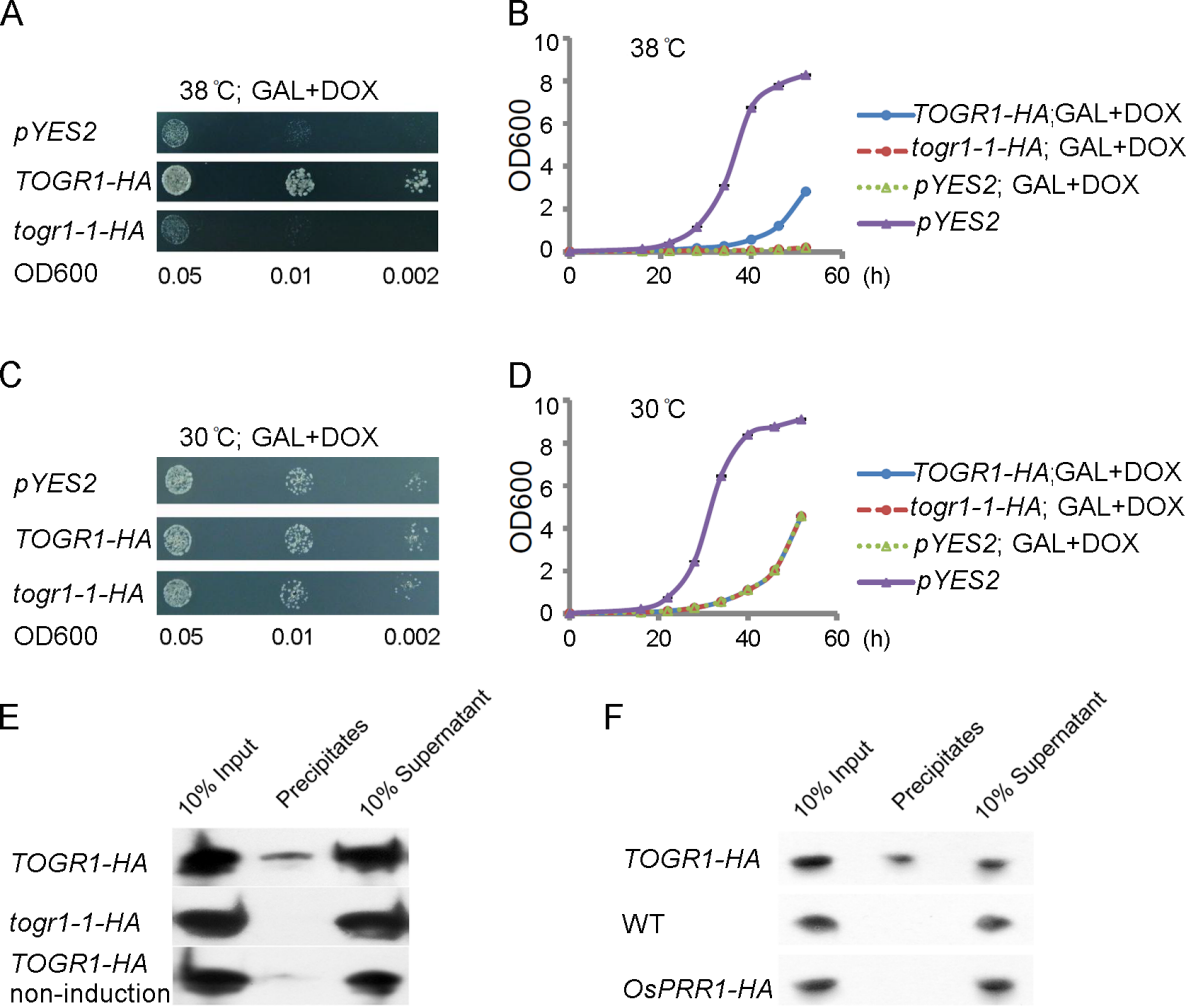
Association of TOGR1 with the SSU is required for an effective cell proliferation at high temperatures. **(A-D)** Complementation of genetically depleted *Rrp3* in yeast by ectopically expressing *TOGR1-HA* or *togr1-1-HA*. A yeast YPH499 strain in which the chromosomally encoded *Rrp3* was placed under control of the doxycycline (DOX)-repressible *tetO7* promoter was transformed with *TOGR1-HA* and *togr1-1-HA* controlled by the galactose (GAL)-inducible promoter *GAL1*. Growth of yeast was monitored on GAL+DOX medium at 38°C and 30°C. A strain carrying empty *pYES2* vector was used as a negative control. Three 10-fold serial dilutions were spotted. For (B and D), data are presented as mean ± SEM (n = 3 replicates). **(E)** Immunoprecipitation-northern assay to detect association of TOGR1-HA and togr1-1-HA with the yeast U3 snoRNA. Uninduced *GAL1*::*TOGR1*:*HA* was used as a negative control. **(F)** Immunoprecipitation-northern assay to detect association of TOGR1-HA with the rice U3 snoRNA *in planta*. Leaf blades of *togr1-1* transformed with *UBi-1*::*TOGR1*:*HA*, wild-type Zhongxian 3037 and Zhonghua-11 transformed with *UBi-1*::*OsPRR1*:*HA* were used.

Regarding that no apparent reduction of the helicase activities was detected for togr1-1, we investigated other potential functional differences between TOGR1 and togr1-1 using budding yeast. It is known that the genetic depletion of *Rrp3* in yeast results in a repressed proliferation [14]. We expressed C-terminal-HA-tagged TOGR1 and togr1-1 (S12 Fig) under the control of a galactose-inducible promoter in an *Rrp3* conditionally depleted yeast strain and found that TOGR1-HA but not togr1-1-HA partially rescued the repressed cell proliferation at 38°C (Fig 4A and 4B), whereas no complementation effects were detected for both TOGR1-HA and togr1-1-HA at 30°C (Fig 4C and 4D), suggesting that TOGR1 but not togr1-1 functions similarly as Rrp3 at high temperatures and further supports the notion that TOGR1 elevates its activities following the temperature increase. It was also revealed that proliferation of the *Rrp3* strain is sensitive to high temperature (Fig 4B and 4D), similar to the *togr1-1* rice mutant. Considering that Rrp3 is recruited to SSU by its central component, the U3 snoRNA [13,14], we next investigated the association of TOGR1-HA and togr1-1-HA with the yeast U3 snoRNA using those transgenic yeast strains. Anti-HA antibodies were used to immunoprecipitate the HA tagged protein from cell lysates. RNA isolated from pellets was analyzed by northern blot with an RNA probe specific to the U3 snoRNA. As controls, immunoprecipitations were also performed on transgenic cells with no induction of *TOGR1-1* expression. As expected, TOGR1-HA prominently coimmunoprecipitated the yeast U3 snoRNA, while togr1-1-HA exhibited no apparent association (Fig 4E), showing that TOGR1 is an RNA helicase being tethered to SSU and the togr1-1 has a defective ability to be recruited to the SSU processome. Association of TOGR1 with the U3 snoRNA was further examined in rice. As expected, anti-HA antibodies immunoprecipitated the U3 snoRNA (Fig 4F) in the *TOGR1-HA* (S12 Fig) plants, while no signal was detected in WT and *OsPRR1-HA* controls, indicating that TOGR1 is associated with the U3 snoRNA and subsequently with SSU in plants.

**Fig 5:**
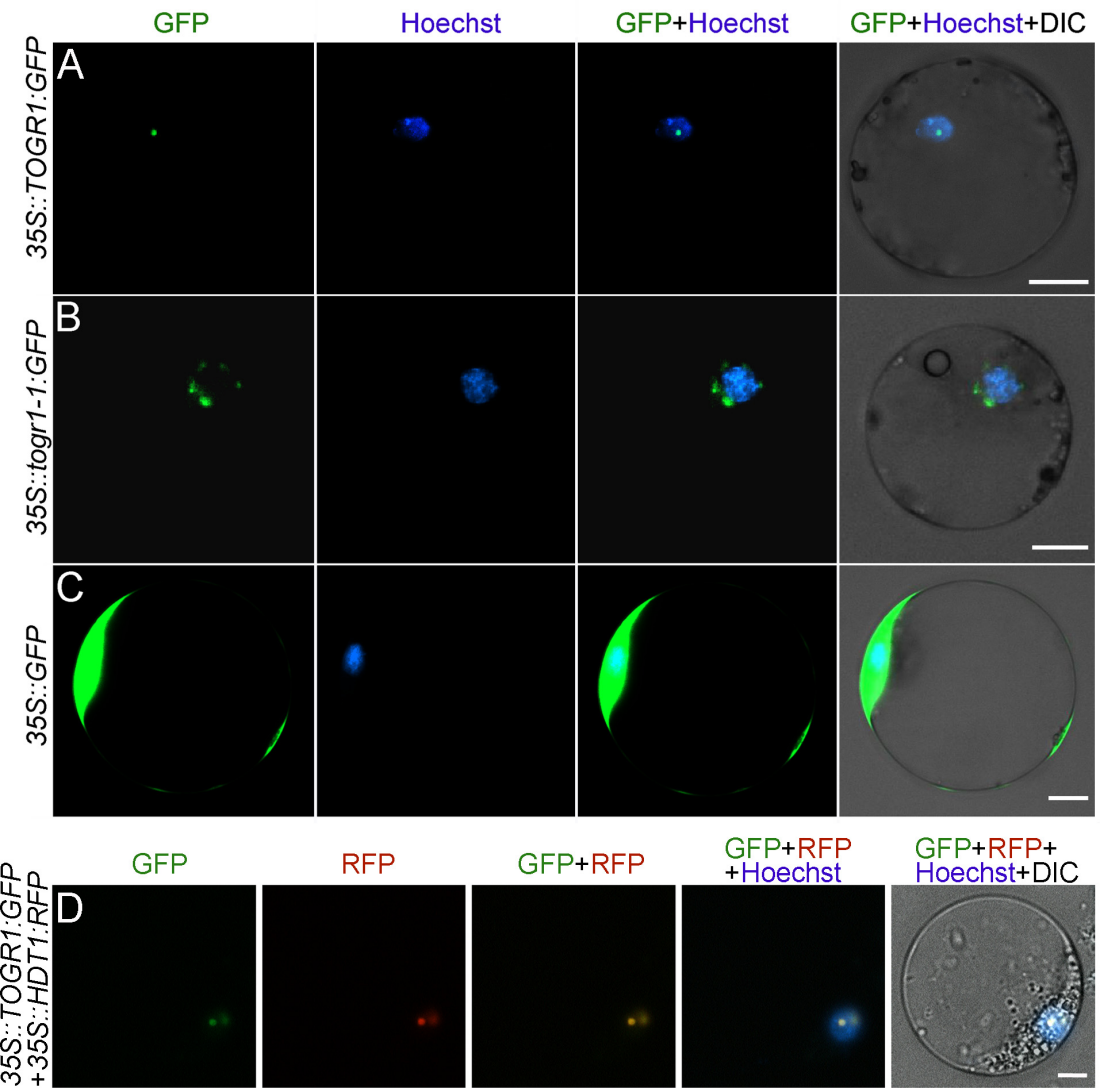
TOGR1 is predominantly localized in the nucleolus, whereas togr1-1 cannot enter the nucleus. Subcellular localization of TOGR1-GFP (A, green) and togr1-1-GFP (B, green) were visualized and photographed in protoplasts. The nucleus was stained by Hoechst dye (purple). HDT1-RFP (D, red) was used as a nucleolus marker. Protoplasts were prepared from rice seedlings and were transformed with constructs containing *35S*::*TOGR1*:*GFP* (A), *35S*::*togr1-1*:*GFP* (B) and *35S*::*TOGR1*:*GFP* plus *35S*::*HDT1*:*RFP*, respectively. *35S*::*GFP* (C) was used as a control. Scale bars: 10 μm.

To characterize the subcellular localization of TOGR1 and togr1-1, green fluorescent protein (GFP) was fused to the C-terminals of TOGR1 and togr1-1, and transiently expressed in protoplasts prepared from rice seedlings under the control of a cauliflower mosaic virus *35S* promoter. With Hoechst dye and RFP-tagged *Arabidopsis* protein HDT1 [37] indicating the nuclear and nucleolar localization, respectively, it was revealed that TOGR1-GFP accumulated in the nucleus and predominantly in the nucleolus (Fig 5A and 5D). By contrast, togr1-1-GFP is only distributed in discrete spots surrounding the nucleus as it was expressed alone in protoplasts (Fig 5B), indicating that togr1-1 has lost the ability being transported into the nucleus. Intriguingly, when togr1-1-GFP was coexpressed with HDT1-RFP, it was co-localized with the marker in the nucleolus (S13A Fig), suggesting that a possible interaction between the two proteins caused an artificial location of togr1-1. Further experiments showed that locations of togr1-2-GFP, togr1-3-GFP and togr1-4-GFP were all similar to TOGR1-GFP (S13B–S13G Fig), indicating that these mutant proteins have other malfunctions rather than abnormal localization.

Further experiments revealed that neither location of TOGR-GFP nor that of togr1-1-GFP is obviously affected by temperature (S14 Fig). It is well established that the primary function of nucleolus is ribosome biogenesis, in which rRNAs are transcribed, processed and assembled with ribosomal proteins to form immature ribosomes [38]. At the early stage of pre-rRNA processing, the U3 snoRNA and its associated proteins are assembled to form the SSU processome onto the growing 35S pre-rRNA chain co-transcriptionally [13], corresponding to the “terminal balls” at the 5' ends of the nascent pre-rRNA transcripts observed in Miller spreads under electron microscopes [38–40]. This is crucial for early pre-rRNA cleavage [41]. Hence, in agreement with the association of TOGR1 with the SSU and the defect of togr1-1 in this, the subcellular localizations of TOGR1 and togr1-1 explain the disordered rRNA maturation of the mutant, and thus further support the idea that TOGR1 is involved in pre-rRNA processing.

The capability of TOGR1 unwinding randomly synthesized RNA duplex indicates its non-sequence specificity, akin to many other RNA helicases [31,42]. This is in contrary with their specialized functions in various biological processes [31,43]. Thus, RNA helicases are generally tethered with their target RNAs or RNP complexes to execute a specific function [10,43]. The mutation point in togr1-1 is a glycine in the N-terminal flanking sequence. This glycine is widely conserved in the homologues of TOGR1 in all analyzed species except yeast Rrp3 (S8 Fig), suggesting its importance in addition to the well defined nine motifs. The togr1-1 protein has lost neither the helicase activities nor the sensitivity to temperature, but rather has lost the competence in being transported into the nucleus and tethered to SSU in the nucleolus, underscoring the critical role of the glycine allowing TOGR1 to be distributed to its functional site and the importance of tethering an RNA helicase to its target.

### TOGR1 Confers Enhanced Plant Growth under Hot Conditions

**Fig 6:**
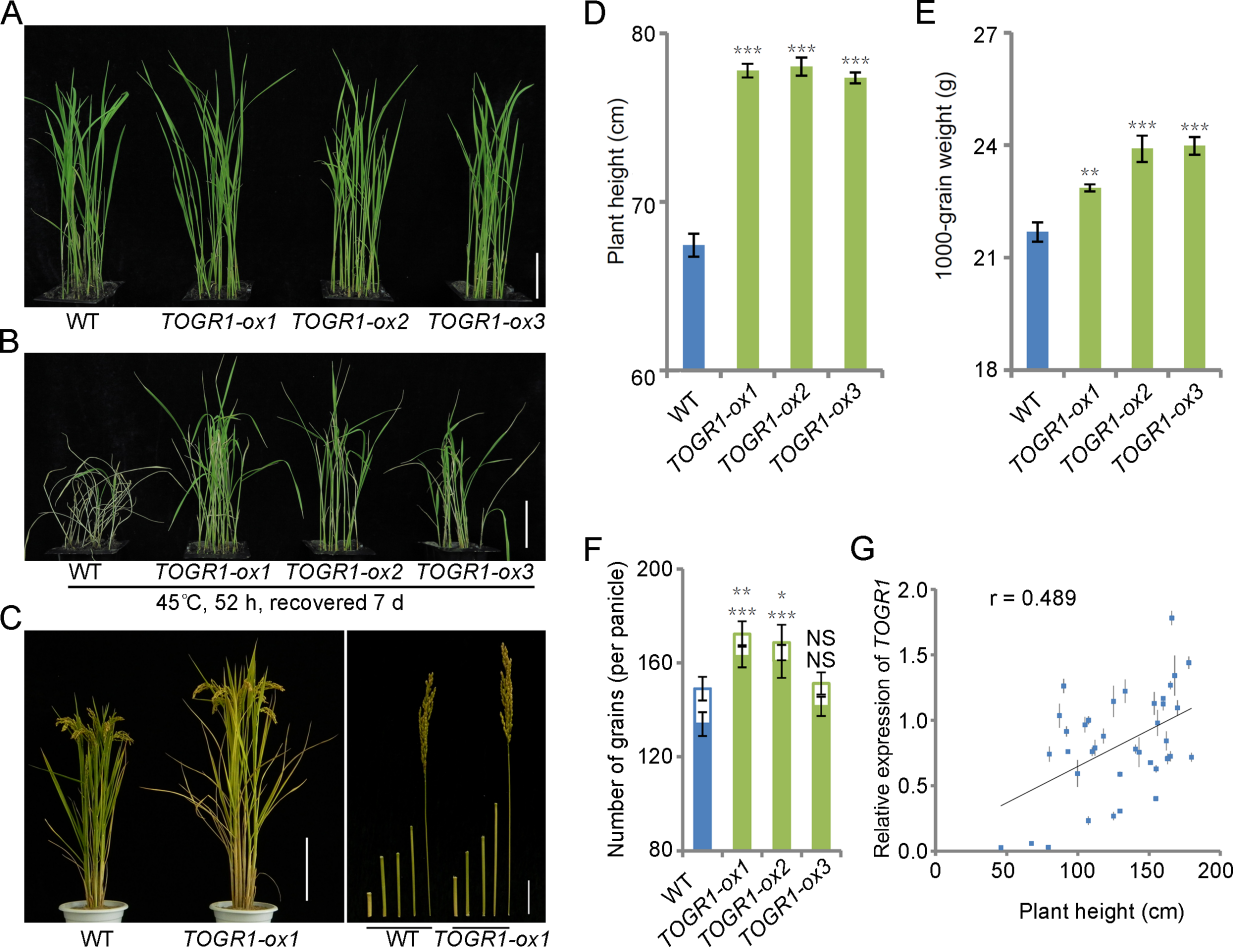
*TOGR1* confers thermotolerant growth under hot weathers. (A and B) Two-week-old WT Yandao 8 and *TOGR1*-overexpressing (*TOGR1-ox*) seedlings grown under 12-h-25°C/12-h-20°C condition were kept at the same condition (A) or were transferred to 45°C for 52 hours and subsequently recovered under 12-h- 25°C/12-h- 20°C for 7 days (B). (C) WT Yandao and *TOGR1*-overexpressing (*TOGR1-ox*) plants grown in Beijing’s summer-autumn fields. Scale bars: plant architecture, 20 cm; internode and panicle, 5 cm. (D-F) Comparison of plant height (B), 1000-grain weight (C) and number of grains per panicle (D) between the WT and *TOGR1*-overexpressing plants. (G) Correlation between plant height and *TOGR1* transcript level in 38 rice accessions including *O*. *sativa*, *O*. *granulata* and *O*. *rufipogon*. Plants were grown in Beijing’s summer-autumn field. RNA was extracted from leave blades and analyzed by qRT-PCR. Data are represented as mean ± SEM (D, n = 30 plants; E, n = 5 replicates; F, n = 30 panicles; G, n = 3 replicates). For (D-F), asterisks indicate statistical significance compared to WT: NS, not significant; *p < 0. 05; **p < 0. 01; ***p < 0.001; one-way ANOVA with *a priori* contrasts. For (G), r = 0.489; p < 0.01; Pearson product-moment correlation coefficient.

To examine effect of elevated *TOGR1* expression on plant thermotolerance, we overexpressed *TOGR1* under control of its native promoter in a rice variety Yandao 8 (S15A–S15C Fig). Two-week-old seedlings grown under 12-h-25°C/12-h-20°C condition were kept at the same condition or subjected to 45°C for 52 hours and subsequently recovered under 12-h-25°C/12-h-20°C for 7 days (Fig 6A and 6B). The transgenic plants showed significantly enhanced thermotolerance than WT after a heat stress treatment, whereas no remarkable difference between them was detected under a cool condition. They were also grown in a paddy field under Beijing’s hot summer-autumn conditions (S15F Fig). In comparison with the WT, the *TOGR1*-overexpressing plants exhibited a remarkable enhancement in plant height, 1000-grain weight and number of grains per panicle (Fig 6C–6F), whereas panicle length and number of panicles per plant were not significantly affected (S15D and S15E Fig). An exception in the number of grains per panicle was that *TOGR1-ox3* had no significant increase like the other two transgenic lines. A possible reason for this is that insertion of T-DNA during transformation disrupted the function of a gene that has positive effect on seed setting and this disruption was then compensated by overexpression of *TOGR1*.

Consistently, qRT-PCR analysis of leaf blades from a collection of 38 rice varieties of *O*. *sativa*, *O*. *granulata* and *O*. *rufipogon* grown under Beijing’s hot summer-autumn conditions revealed that plant height has a moderate positive correlation (r = 0.489; p < 0.01) with the expression level of *TOGR1* (Fig 6G). These results strongly support the idea that an enhancement of *TOGR1* expression improves plant growth under hot conditions.

### TOGR1 Mediates Adaptation of the Primary Metabolism to High Temperatures

**Fig 7:**
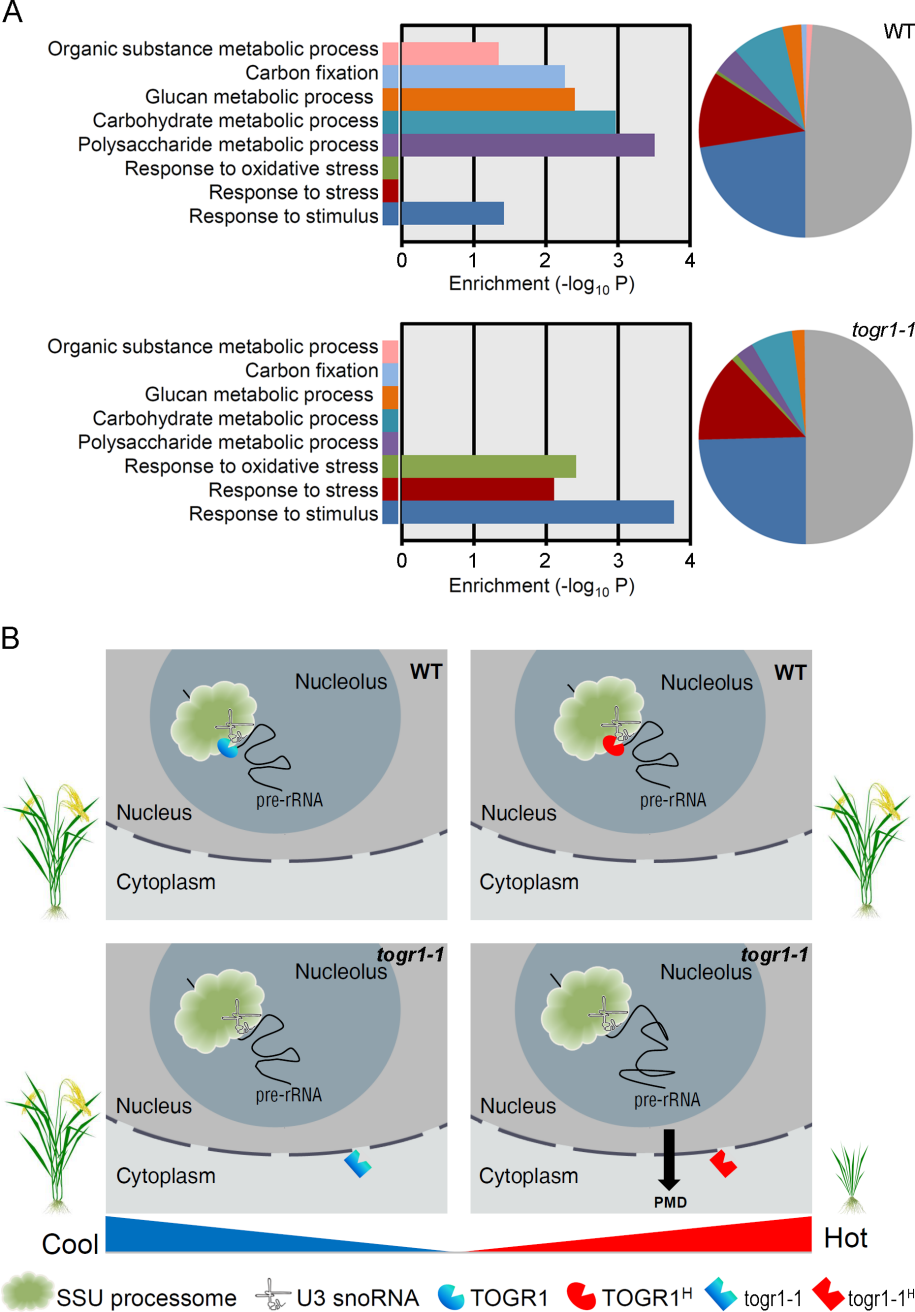
TOGR1 coordinates the primary metabolic processes required for plant growth at high temperatures. **(A)** GO functional enrichment analysis of differentially expressed genes (DEGs, ≥ 2-fold changes) in WT and *togr1-1* seedlings following a temperature increase from 25°C to 30°C, respectively. The pie charts show the frequency of the GO terms in total DEGs. The bar charts show enrichment levels of the GO terms comparing to the genome background. Significantly enriched GO terms with p value < 0.05 are shown. Ultra-geometric test was used. **(B)** Schematic diagram showing that TOGR1 serves as an intrinsic pre-rRNA processing chaperone in the nucleolus in a temperature-dependent manner. At high temperatures, proper and effective pre-rRNA processing in plants requires pre-rRNAs in a correct conformation. Being recruited to the SSU within the nucleolus and with the expression and enzymatic activity enhanced by temperature increase, TOGR1 aids the processing of pre-rRNAs by remodeling RNA conformation and allowing a proper RNP- RNA interaction, leading to an effective rRNA biogenesis required for coherent coordination of primary metabolic processes and plant growth. By contrast, togr1-1 has lost the ability of being transported into the nucleolus and is unable to remodel misfolded rRNA precursors, leading to an improper pre-rRNA processing and thus a primary metabolic disorder (PMD) and retarded plant growth at high temperatures. TOGR1^H^ and togr1-1^H^ represent proteins with elevated activities in response to temperature increase.

In order to investigate the impact of rRNA biogenesis disorder on transcriptome, we performed RNA sequencing in three-week-old rice seedlings grown in chambers at 25°C and 30°C, respectively. It was shown that when temperature increases, both the wild-type and *togr1-1* dramatically adjust the expression levels of more than 2,000 genes (S16 Fig). Gene ontology (GO) enrichment analysis of the differentially expressed genes (DEGs) in the wild-type revealed several significantly enriched biological process categories required for growth including polysaccharide metabolic process (GO:0005976), carbohydrate metabolic process (GO:0005975), glucan metabolic process (GO:0044042), carbon fixation (GO:0015977) and organic substance metabolic process (GO:0071704), in addition to response to stimulus (GO:0050896; Fig 7A; S3 Table). By contrast, significantly enriched biological processes in *togr1-1* contained response to stimulus, response to oxidative stress (GO:0006979) and response to stress (GO:0006950), whereas the coordination of primary metabolic processes and carbon fixation were largely impaired or undetectable (Fig 7A; S3 Table). Under a moderate high temperature (30°C) that is known to promote growth of rice rather than cause heat stress [29], *togr1-1* appears to primarily divert resources to cope with environmental stimulus rather than to support growth, which normally occurs when plants are exposed to heat-stress conditions [44–46].

It was reported that ascorbate peroxidase 2 (APX2) and galactinol synthase 2 (GolS2) both protect plants from heat stress by scavenging of ROS (reactive oxygen species) and producing of osmoprotectants respectively [47–49]. To study if TOGR1’s function is related to these two genes, we next examined transcript levels of homologues of *APX2* (*LOC_Os07g49400*) and *GolS2* (*LOC_Os03g20120* and *LOC_Os07g48830*) in WT rice and *togr1-1*seedlings (S16B Fig). After one-day treatment of 45°C heat stress for seedlings previously grown at 25°C, all of the three genes showed significantly increased expressions in *togr1-1*, whereas those in the WT either not significant or the increased levels much lower. This reflects a much stronger up-regulation of the three genes in response to heat stress in *togr1-1* than that in the WT, and thus indicates a compensation to the internal disorders caused by dysfunction of *TOGR1*, but does not support the hypothesis that TOGR1 functions in the same pathway with *APX2* and *GolS2*.

Taken together, these results suggested that TOGR1 is required for a primary metabolism adaptation to at high temperatures and its mutation results in a system disorder impeding this adaptive mechanism required for normal cell division and thus plant growth at high temperatures.

## Discussion

As a ribosome factory, the nucleolus has been reported to be sensitive to various stresses including heat [50,51]. It has been proposed that during the early stage of pre-rRNA processing, the nascent pre-rRNA transcript might wrap around the SSU processome which mediates early stage cleavages [52]. Thus an effective processing requires proper RNA foldings and RNA-protein interactions regarding that RNA conformation is vulnerable to temperature changes [53]. In fact, the nucleolus contains numerous non-ribosomal proteins associated with the SSU processome to facilitate RNA folding and interaction of many components of it [54]. A number of them have been identified as RNA helicases [13,14]. Members of this protein family are known to protect organisms from various abiotic stresses including cold stress tolerance [9], but little is known about their roles in at high temperature tolerance. Our study revealed that, an SSU-associated nucleolar RNA helicase, TOGR1, appears to significantly affect early pre-rRNA cleavage (Fig 3) and is essential for the thermotolerance mechanism of rice growth. This is mediated by priming pre-rRNA processing in response to ambient temperature increase as an RNA chaperone (Fig 7B). At cool temperatures, likely with the help of other RNA helicases and the role of TOGR1 almost dispensable, the pre-rRNAs are in a functional conformation that can be readily processed into mature rRNAs to support plant growth. However, at high temperatures, the pre-rRNAs are misfolded and the interactions between pre-rRNAs and its processing proteins are disrupted, which would result in an ineffective processing of pre-rRNAs if without the assistance of TOGR1. Being recruited to the SSU in the nucleolus and with its expression and activity enhanced by temperature increases, TOGR1 appears to aid pre-rRNAs to form native conformation and proper RNA-protein interactions as an RNA chaperone. This action thus ensures the production of normal amount of rRNAs required for a proper coordination of primary metabolisms and normal plant growth at high temperatures. By contrast, the dysfunction of togr1-1 in its inability of being transported to the nucleolus and thus recruited to SSU makes it unavailable when rRNA biogenesis needs a protection from high temperature leading to pre-rRNA processing and primary metabolism disorders and thus retarded plant growth. Up to date, this is the only plant RNA helicase that has been found to modulate the growth thermotolerance, revealing a novel molecular mechanism for plants to acclimate to hot weathers.

Ribosomal RNA biogenesis is crucial for eukaryotic growth control [55,56], and is tightly coupled to environmental changes [57]. Our results demonstrate that rice enhances the rate of rRNA biogenesis at high temperatures. However, temperature increase also leads to a disruption of RNA homeostasis which further causes the delayed pre-rRNA processing, similar to the well-established cold stress effect [9,18,19,21]. Thus, RNA helicases are needed to support an effective processing of pre-rRNA at high temperatures. The retarded rRNA biogenesis and plant growth defects exhibited by the *togr1-1* mutant at high temperatures indicate an indispensable role of TOGR1 as an RNA chaperone in aiding pre-rRNA processing and normal plant growth as ambient temperature increasing.

Plants sense ambient temperature change via a set of primary thermosensors including membrane localized Ca^2+^ channels [6] and H2A.Z containing nucleosomes [7]. All provide signals for plants to make internal adjustment to adapt to temperature fluctuation. To protect pre-rRNA processing from high temperature, *TOGR1* appears to be up-regulated by temperature increasing signal and, in addition, its expression is regulated by the circadian clock and thus is synchronized with a daily temperature fluctuation which can be anticipated by the circadian clock. Our findings provide a remarkable example of high temperature induced RNA helicases, revealing a new role for a member of this protein family and, in particular, paving a way for studying the roles of RNA helicase and rRNA biogenesis in plant thermotolerance. TOGR1 appears to serve as a critical element of a “buffering” system against unfavourable temperature conditions for plants. That means this protein makes plants adaptive to a more broad range of temperatures by conferring thermotolerance. Nevertheless, further details about temperature sensing pathway of this system remain to be elucidated.

Our study has also established a molecular connection between rRNA biogenesis and cell division. It has been argued that ribosome biogenesis is a key component of the signaling network controlling cell growth and division and play roles in specific aspects of development and physiology rather than a general growth control [55,56,58]. TOGR1 may participate in a coordination program of primary metabolisms via its effect on rRNA biogenesis. Coordinated primary metabolisms and sufficient rRNA availability facilitated by TOGR1 probably provide both signals and material basis for proper cell division at high temperatures. This is consistent with a previous report that mutation of 3 ribosome biogenesis genes in *Arabidopsis* caused the similar effect [58]. However, the pathways that ribosome biogenesis influences cell division remains to be explored in more detail.

In conclusion, our results demonstrate that TOGR1 functions as a thermosensitive RNA chaperone in the nucleolus to mediate normal plant growth at high temperatures, providing a novel insight into the endogenous mechanisms protecting plants from hot weathers and a possibility to mitigate their adverse impacts on plant productivity.

## Materials and Methods

### Plant Materials and Growth Conditions

The *togr1-1* mutant was isolated from a rice (*Oryza sativa* L. ssp. *indica*) variety Zhongxian 3037. Plants were grown in the paddy fields under natural conditions or in growth chambers (12L:12D cycle with a light intensity of 200 μmol quanta m^-2^ s^-1^ and 80% humidity, unless otherwise specified). For circadian analysis, two-month-old Zhongxian 3037 plants were first entrained under 12L:12D conditions for 10 days and then transferred to continuous light conditions, and temperature was kept at 30°C. Details of positional cloning, transgenic plants and plasmid constructions were described in S1 Text.

### TILLING Screening

Allelic mutants were obtained from http://croptilling.org/ and were screened out using a previously described TILLING method [59]. Primer pairs *TOGR1T1F/ TOGR1T1R* and *TOGR1T2F/ TOGR1T2R* (S1 Text) were used for amplification.

### Tissue Stain

Cross-sections of internodes, leaf blades and roots were stained by both safranin and fast green, and leaf sheaths were stained by safranin (S1 Text).

### GUS Histochemical Analysis

A Construct containing *TOGR1pro*::*GUS* was transformed into rice, and the transgenic plants were analyzed with a GUS staining assay as described previously [60].

### Phylogenetic Analysis

The protein sequences homologous to TOGR1 were found by BLASTp (http://www.ncbi.nlm.nih.gov/BLAST/) using the entire amino acid sequence of TOGR1 as a query. The obtained amino acid sequences were aligned using Clustal W software [61] (http://www.ebi.ac.uk/clustalw/) and shaded using GenDoc. A rooted phylogenetic tree was constructed using the neighbor-joining method of the MEGA 5.2 software [62] with the following parameters: Poisson model, pairwise deletion, and bootstrap (1000 replicates).

### Subcellular Localization of the GFP Fusion Proteins

The fusion constructs (*35S*::*TOGR1*:*GFP* and *35S*::*togr1-1*:*GFP*) and control (*35S*::*GFP*) were transiently expressed in protoplasts prepared from etiolated rice seedlings as described [63]. *35S*::*HDT1*:*RFP* was used as a nucleolar marker. Protoplasts were stained by Hoechst 33258 (Sigma-Aldrich) before visualization and photography; see S1 Text.

### Helicase Assay

The 3′-overhang RNA duplex used for helicase assay were arbitrarily designed to minimize secondary structures [64] and labeled at the 5′ end of the indicated strand with ROX fluorophore (Fig 2E; Takara Bio). The ROX labeled upper strand was used as a marker and the same strand without labeling was used as a trap for the displaced lower strand of the RNA duplex. Helicase assay was performed on purified proteins using a method modified from previously described [64]. Detailed procedures of protein expression and purification and helicase assay are provided in S1 Text.

### Yeast Growth Assays

For complementation analysis, YPH499 (*MAT*a *ura3-52 lys2-80 ade2-101 trp1-Δ63 his3-Δ200 leu2-Δ1*) in which the chromosomally encoded *Rrp3* was placed under the control of the DOX-repressible *tetO7* promoter was used as parent strain [14]. Strain carrying empty *pYES2* vector or *pYES2* plasmid containing *GAL1*::*TOGR1*:*HA* or *GAL1*::*togr1-1*:*HA* was spotted on induction medium containing both DOX and D-galactose in a three 50-fold serial dilutions at OD600 0.5, 0.01 and 0.002. Growth curves were monitored in liquid induction medium, and strain carrying empty *pYES2* grown in liquid synthetic minimal medium was used as control (S1 Text).

### Immunoprecipitation

For rice, *UBi-1*::*TOGR1*:*HA* plants in *togr1-1* background were grown for 60 days in growth chamber. Wild-type Zhongxian 3037 and *UBi-1*::*OsPRR1*:*HA* plants in Zhonghua-11 background were used as negative controls. Leaf blades were harvest and plant whole-cell extracts were prepared as previously described [65].

For yeast, *tetO7*::*Rrp3* YPH499 strain harbouring *GAL1*::*TOGR1*:*HA* or *GAL1*::*togr1-1*:*HA* were cultured in liquid induction medium containing both DOX and D-galactose at 30°C. The *GAL1*::*TOGR1*:*HA* strain and the parent strain grown in liquid synthetic minimal medium were used as negative controls. At exponential stage, yeast whole-cell extracts were prepared according to a published method [66] using glass beads (Sigma-Aldrich). Immunoprecipitation was performed as previously described [67]. Total RNA was then extracted for northern analysis. Detailed procedures are provided in S1 Text.

### RNA Methods

For northern blotting, RNA probes antisense to the rice or yeast U3 snoRNA labeled with [α-^32^P] UTP and oligonucleotides labeled with [γ-^32^P]ATP at the 5′ ends were used to detect the U3 snoRNAs and pre-rRNA processing intermediates, respectively. The 5′ and 3′ ends of rRNA precursors were determined by circular RT-PCR as described previously [68]. For qRT-PCR, three biological and three technical repeats were performed in the experiments. Primers and detailed procedures are provided in S1 Text.

### Transcriptome Resequencing and Gene Ontology (GO) Analysis

RNA sequencing of Zhongxian 3037 at 25°C and 30°C and *togr1-1* at 25°C and 30°C was conducted by BGI (Shenzhen, China). DEGs (differentially expressed genes) with difference equal or higher than 2-fold between the two temperature levels were then mapped to GO terms in the database (http://www.geneontology.org/). Ultra-geometric test was used to find significantly enriched GO terms in DEGs comparing to the genome background, taking p≤0.05 as a threshold; see S1 Text.

### Protein Gel and Blot Analysis

Protein samples extracted from plant leaves or yeast cells were separated on SDS-PAGE gels, and then directly visualized or analyzed by Western blot (S1 Text).

### Accession Numbers

RNA-seq data have been deposited in the Gene Expression Omnibus (GEO) under accession number GSE42096.

## Supporting Information

S1 Fig Daily maximum and minimum temperatures of rice-growing fields at three locations of China. Brown curves, Beijing from May 13^th^ to Sep 20^th^ (summer-autumn) in 2011; red curves, Yangzhou in the Yangtze River Delta from May 13^th^ to Sep 20^th^ (summer-autumn) in 2011; blue curves, Lingshui in Hainan Island, from Dec 1^st^, 2010 to Apr 10^th^ (winter-spring), 2011.
(TIF)

S2 Fig Phenotypic characterization of the *togr1-1* mutant. (A-E) Comparison of plant height, number of panicles per plant, number of grains per panicle, panicle length and 1000-grain weight between WT and *togr1-1* grown in Beijing and Lingshui’s fields. Filled and unfilled columns in (C) indicate numbers of filled and unfilled grains, respectively. (F-H) Evaluation of thermosensitivity of *togr1-1* seedlings. Newly geminated WT and *togr1-1* seedlings were grown in climate chambers at indicated temperatures for three weeks before detecting maximum root length, crown root number and upground plant height. Data are represented as mean ± SEM (A and B, n = 20 plants; C and D, n = 20 panicles; E, n = 5 replicates; F-H, n = 15 plants). Asterisks indicate statistical significance compared to WT: NS, not significant; *p < 0.05; ***p < 0.001; one-way ANOVA with *a priori* contrasts.
(TIF)

S3 Fig Comparison of mature organs between WT and *togr1-1* grown under Beijing’s summer-autumn conditions. (A) Plant architectures of WT (left) and *togr1-1* (right). FL, flag leaf; 15th, the fifteenth leaf; P, panicle; I-V, internode I-V. Scale bar: 5 cm. (B) Cross-sections of the 15th leaf blades of plants. LVB, large vascular bundle; SVB, small vascular bundle; MB, midrib. Scale bars: 0.5 mm. (C-E) Comparison of internode (C, IN), leaf blade (C, LB), root maturation zone (C, RM) and leaf sheath (D and E) cells between the WT and *togr1-1*. Internodes were collected from plants grown in Beijing’s summer-autumn field at heading stage. Leaf blades and sheaths and roots were collected from seedlings grown at 35°C. Scale bars: 50 μm. For leaf sheath, cell length was measured and cell number along the longitudinal direction was counted. For (E), data are represented as mean ± SEM (cell length measurement, n = 80 cells; cell number counting, n = 4 leaf sheaths). Asterisks indicate statistical significance compared to WT: NS, not significant; ***p<0.001; t-test.
(TIF)

S4 Fig Isolation of *TOGR1* gene. (A) Fine mapping and cloning of the *TOGR1* locus. The mutation site at the *togr1-1* genome is indicated by an inverted vertical arrow. *P1* to *P10* are polymorphic DNA markers developed in this work. Numbers of recombinants are shown under each marker. *BAC1*-*BAC8* correspond to *AC151537*, *AC139174*, *AC135792*, *AC145388*, *AC133930*, *AC146718*, *AC105747* and *AC093018*, respectively. (B) Growth of the complemented *togr1-1*+*TOGR1* plant in comparison that of WT and *togr1* in Beijing’s summer-autumn field. A construct containing *TOGR1pro*::*TOGR1* was used for complementation transformation. Scale bar: 15 cm. (C) PCR identification of *TOGR1* complementation transgenic lines. Primers *35SF* and *35SR* were used to confirm the presence of the transgene. Partial 17S rRNA gene amplified by primers *17SF*/*17SR* was used as reference. NC, negative control of non-transgenic plant. Sizes of DNA markers are given on the left.
(TIF)

S5 Fig Allelic mutants of *togr1*. (A and B) Mutation sites of four allelic mutants of *togr1*. *togr1-1* is in Zhongxian 3037 background, and *togr1-2* to *-4* are in Zhonghua 11 background. (C and D) Three-month-old plants grown in a paddy field under Beijing’s summer-autumn conditions. Scale bars: 10 cm. (E) Two-week-old seedlings grown in chamber at 25 and 35°C. Scale bars: 5 cm.
(TIF)

S6 Fig Daily maximum and minimum temperatures of the allelic mutants growing fields in Beijing from May 11^th^ to Jul 31^st^ (summer) in 2015. (TIF)

S7 Fig histochemical analysis of expression of TOGR1. β-Glucuronidase (GUS) histochemical analysis of *TOGR1* expression shows its expression in leaf blade, seedling shoot, roots, internode, and anthers. Plants were transformed with a construct containing *TOGR1pro*::*GUS*. Scale bars: leaf blade, 2 mm; cross-section of leaf blade and internode, 0.1 mm; seedling shoot, roots and spikelet, 5 mm; internode and anther, 1 mm.
(TIF)

S8 Fig Amino acid sequence alignments of TOGR1 protein and its related sequences in 13 species. Amino acid sequences were aligned using Clustal W. Alignment was shaded using GenDoc. Identical amino acid residues and conservative changes are indicated in black and grey background, respectively. Nine conserved motifs are labeled. The mutation site in togr1 is marked with asterisk.
(PDF)

S9 Fig A phylogenetic tree showing TOGR1 and its related sequences in 13 species. The tree was constructed using the neighbor-joining method based on a Clustal W alignment. Bootstrap values based on 1000 replications are indicated in their respective nodes. The scale bar indicates genetic distance based on branch length.
(PDF)

S10 Fig Expression and purification of TOGR1 and togr1 proteins for helicase activity analysis. Desired proteins were cleaved from N-His-SUMO-fusion proteins purified from *E*. *coli* lysates and the GST-tagged protease used for cleavage was removed by using GST binding resin. Proteins were run on 12% polyacrylamide gel.
(TIF)

S11 Fig Sequence of the 17S precursor *P-A3* detected by circular RT-PCR. Sequence of the 17S rRNA region is shown in bold letters.
(PDF)

S12 Fig Western blot analysis of HA-tagged proteins expressed in yeast and rice plants. Lanes 1 and 2, *tetO7*::*Rrp3* yeast strain carrying *GAL1*::*TOGR1*:*HA* and *GAL1*::*togr1*:*HA*, respectively; lanes 3 and 4, *togr1* rice plants carrying *UBi-1*::*TOGR1*:*HA*. Yeast carrying empty *pYES2* (lane 5) and wild-type rice (lane 6) were used as negative controls. Total protein was extracted from yeast or rice leaf blades and run on 12% polyacrylamide gel. HA-tagged proteins were detected using mouse anti-HA monoclonal antibodies. ACTIN was analyzed by anti-ACTIN as an internal control.
(TIF)

S13 Fig Localization of togr1-1-GFP, togr1-2-GFP, togr1-3-GFP and togr1-4-GFP. Subcellular localization of togr1-1-GFP (A, green), togr1-2-GFP (B and E, green), togr1-3-GFP (C and F, green) and togr1-4-GFP (D and G, green) were visualized and photographed in protoplasts. The nucleus was stained by Hoechst dye (purple). HDT1-RFP (red) was used as a nucleolus marker. Protoplasts were prepared from rice seedlings and were transformed with respective constructs. Scale bars: 10 μm.
(TIF)

S14 Fig Localization of TOGR1-GFP and togr1-1-GFP at two different temperature levels. Subcellular localization of TOGR1-GFP (A and B, green) and togr1-1-GFP (C and D, green) were visualized and photographed in protoplasts. The nucleus was stained by Hoechst dye (purple). Protoplasts were prepared from rice seedlings and were transformed with constructs containing *35S*::*TOGR1*:*GFP* (A and B) and *35S*::*togr1-1*:*GFP* (C and D), respectively. Transformed protoplasts were either incubated at 4°C for one day (A and C) or treated with 22h°C 20+ 2h 35°C (B and D). Scale bars: 10 μm.
(TIF)

S15 Fig Over-expressing *TOGR1* in rice plants. (A) WT and *TOGR1*-overexpressing plants at heading stage. Scale bar: 20 cm. (B) PCR identification of *TOGR1*-overexpressing transgenic lines. Primers *35SF* and *35SR* were used to confirm the presence of the transgene. Partial 17S rRNA gene amplified by primers *17SF*/*17SR* was used as reference. NC, negative control of non-transgenic plant. Sizes of DNA markers are given on the left. (C) Transcript level of *TOGR1* in the WT and *TOGR1*-overexpressing transgenic lines. The average transcript level of *TOGR1* in WT is set to 1. (D and E) Comparison of panicle length and number of panicles per plant between the WT and *TOGR1*-overexpressing transgenic lines grown in Beijing’s summer-autumn fields. Data are represented as mean ± SEM (C, n = 3 replicates; D, n = 30 plants; E, n = 120 panicles). Asterisks indicate statistical significance compared to WT: NS, not significant; **p < 0.01; ***p < 0.001; one-way ANOVA with *a priori* contrasts. (F) Daily maximum and minimum temperatures of the rice-growing fields in Beijing from May 13^th^ to Sep 20^th^ (summer-autumn) in 2014.
(TIF)

S16 Fig Analysis of differentially expressed genes (DEGs) in *togr1* and WT plants at different temperatures. (A) Wild-type and *togr1* seedlings were grown at 25°C (WT-25°C and *togr1*-25°C) and 30°C (WT-30°C and *togr1*-30°C) for 18 days, respectively. Total RNA was extracted from leaf blades. Transcriptome data were obtained from RNA sequencing. Numbers of DEGs between samples are compared as indicated. (B) Transcript levels of *LOC_Os07g49400*, *LOC_Os03g20120* and *LOC_Os07g48830* in the WT and *togr1-1* seedlings after one-day of 45°C heat stress treatment. Seedlings grown at 25°C were used as controls. The average transcript levels of the three genes in WT at 25°C are set to 1. Data are represented as mean ± SEM (n = 3 plants). Asterisks indicate statistical significance: NS, not significant; *p < 0.05; **p < 0.01; ***p < 0.001; one-way ANOVA with *a priori* contrasts.
(TIF)

S1 Table List of the oligonucleotides used for northern analysis and circular RT-PCR. (PDF)

S2 Table Relative transcript level of *TOGR1* in 38 rice accessions. (PDF)

S3 Table GO functional enrichment analysis of differently expressed genes (DEGs, ≥2-fold change) following a temperature increase in WT and mutant (*togr1*) seedlings. (PDF)

S1 Text Supplemental experimental procedures. (PDF)
