# Supplementary figures and images for: Nucleolar DEAD-Box RNA Helicase TOGR1 Regulates Thermotolerant Growth as a Pre-rRNA Chaperone in Rice
Source: PLoS Genet. 2016 Feb 5;12(2):e1005844. doi: 10.1371/journal.pgen.1005844 (PMC4743921; doi:10.1371/journal.pgen.1005844)

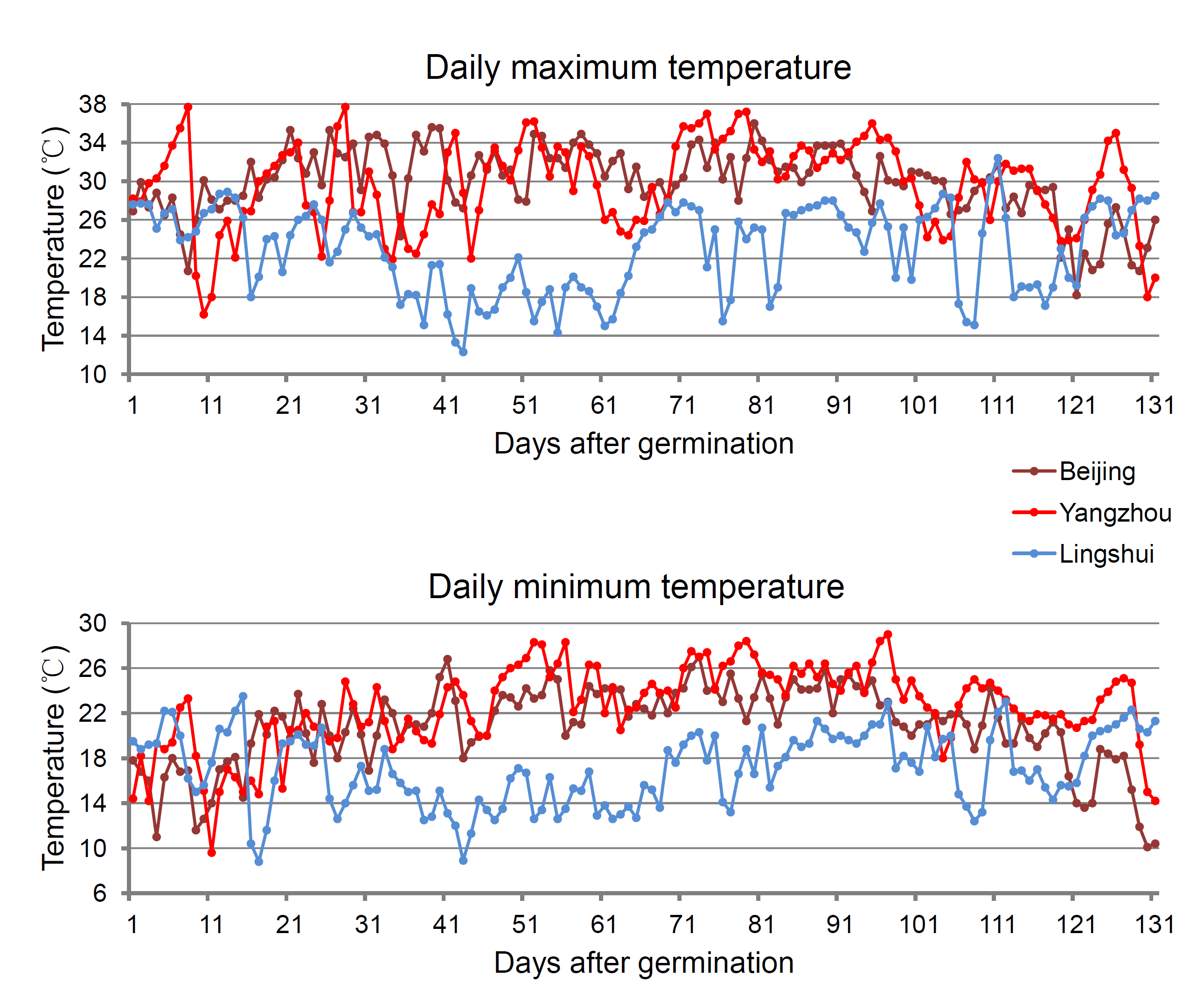

Supplement: S1 Fig — Brown curves, Beijing from May 13th to Sep 20th (summer-autumn) in 2011; red curves, Yangzhou in the Yangtze River Delta from May 13th to Sep 20th (summer-autumn) in 2011; blue curves, Lingshui in Hainan Island, from Dec 1st, 2010 to Apr 10th (winter-spring), 2011. (TIF) [file pgen.1005844.s001.tif]

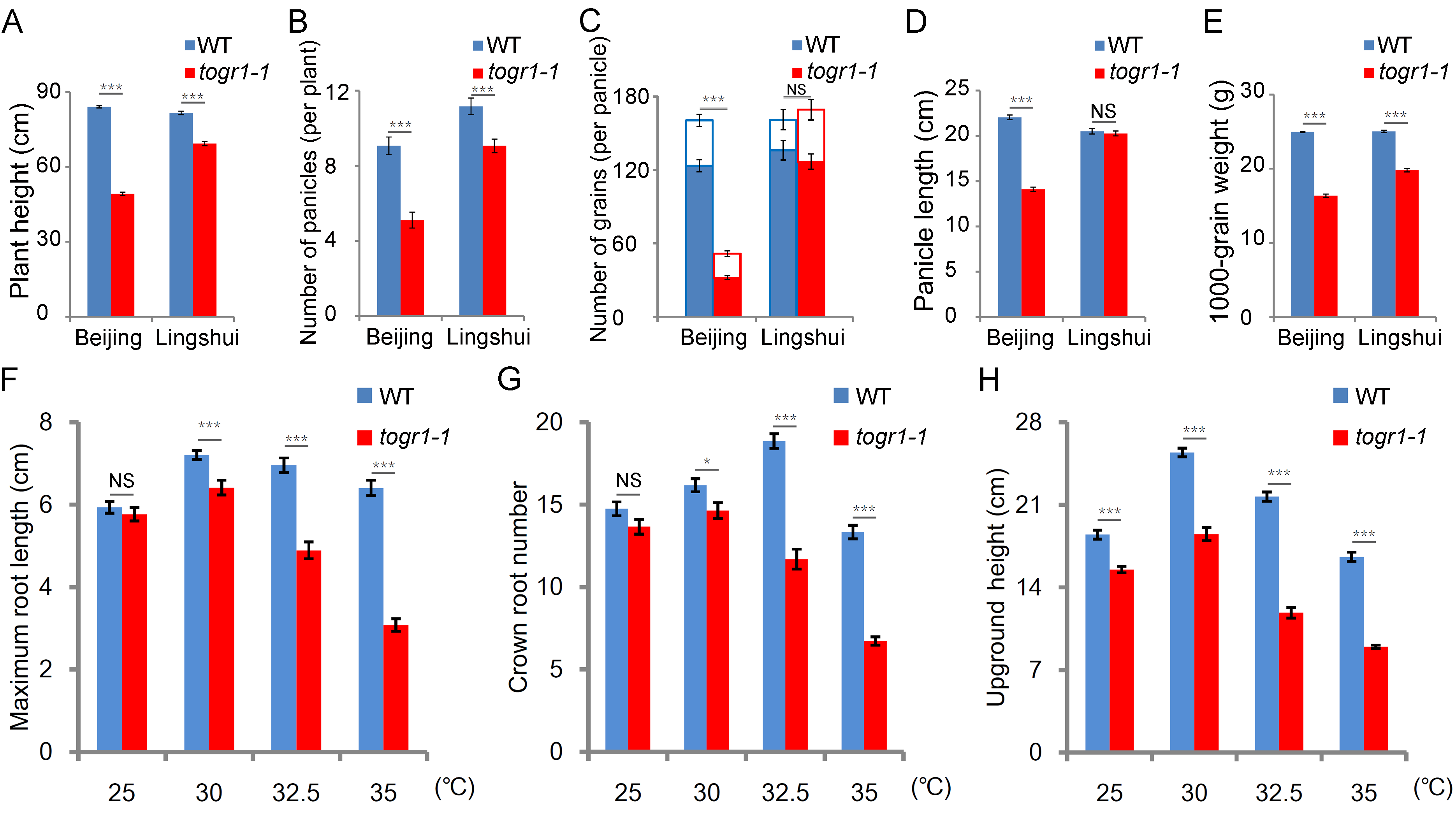

Supplement: S2 Fig — (A-E) Comparison of plant height, number of panicles per plant, number of grains per panicle, panicle length and 1000-grain weight between WT and togr1-1 grown in Beijing and Lingshui’s fields. Filled and unfilled columns in (C) indicate numbers of filled and unfilled grains, respectively. (F-H) Evaluation of thermosensitivity of togr1-1 seedlings. Newly geminated WT and togr1-1 seedlings were grown in climate chambers at indicated temperatures for three weeks before detecting maximum root length, crown root number and upground plant height. Data are represented as mean ± SEM (A and B, n = 20 plants; C and D, n = 20 panicles; E, n = 5 replicates; F-H, n = 15 plants). Asterisks indicate statistical significance compared to WT: NS, not significant; *p < 0.05; ***p < 0.001; one-way ANOVA with a priori contrasts. (TIF) [file pgen.1005844.s002.tif]

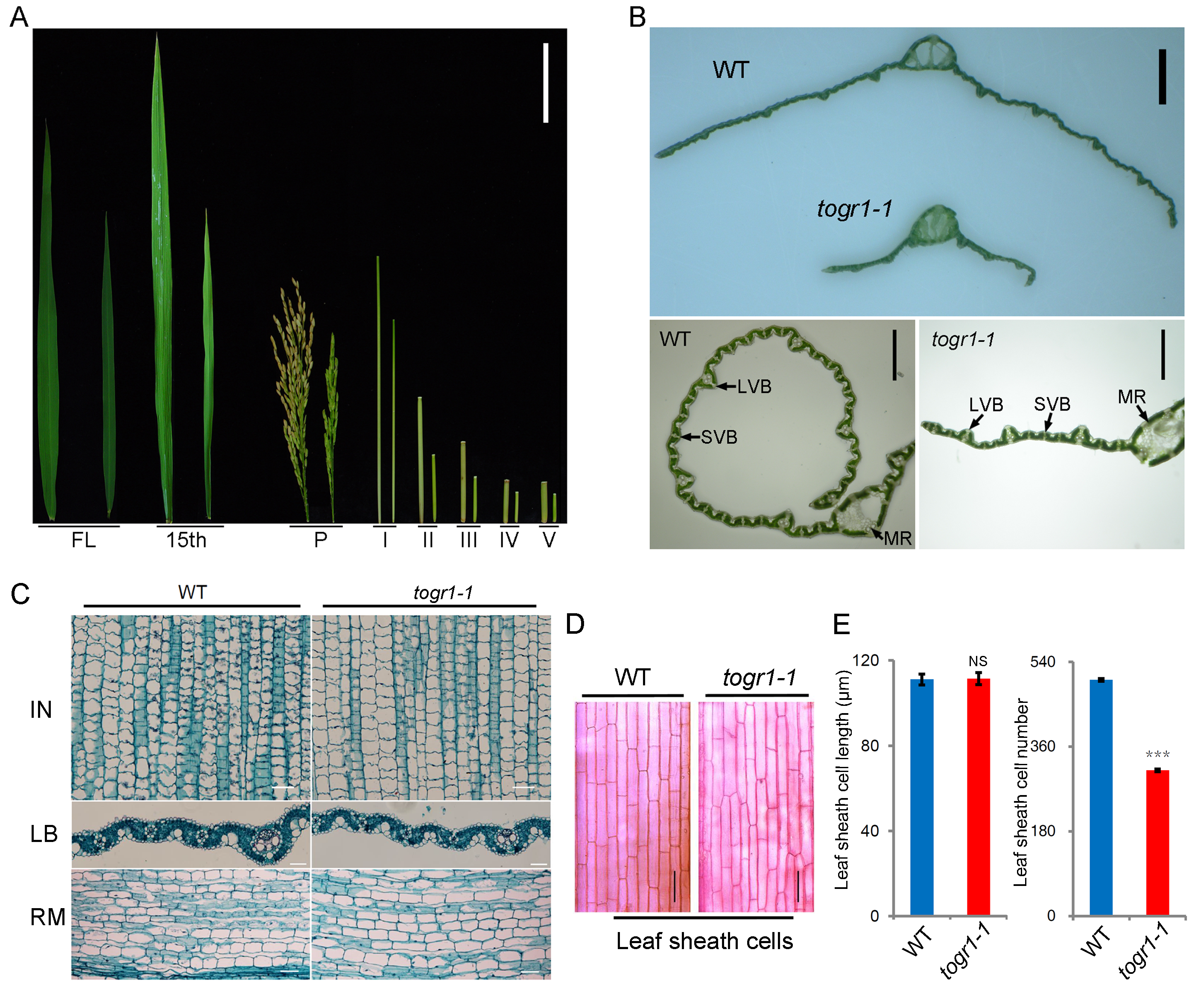

Supplement: S3 Fig — (A) Plant architectures of WT (left) and togr1-1 (right). FL, flag leaf; 15th, the fifteenth leaf; P, panicle; I-V, internode I-V. Scale bar: 5 cm. (B) Cross-sections of the 15th leaf blades of plants. LVB, large vascular bundle; SVB, small vascular bundle; MB, midrib. Scale bars: 0.5 mm. (C-E) Comparison of internode (C, IN), leaf blade (C, LB), root maturation zone (C, RM) and leaf sheath (D and E) cells between the WT and togr1-1. Internodes were collected from plants grown in Beijing’s summer-autumn field at heading stage. Leaf blades and sheaths and roots were collected from seedlings grown at 35°C. Scale bars: 50 μm. For leaf sheath, cell length was measured and cell number along the longitudinal direction was counted. For (E), data are represented as mean ± SEM (cell length measurement, n = 80 cells; cell number counting, n = 4 leaf sheaths). Asterisks indicate statistical significance compared to WT: NS, not significant; ***p<0.001; t-test. (TIF) [file pgen.1005844.s003.tif]

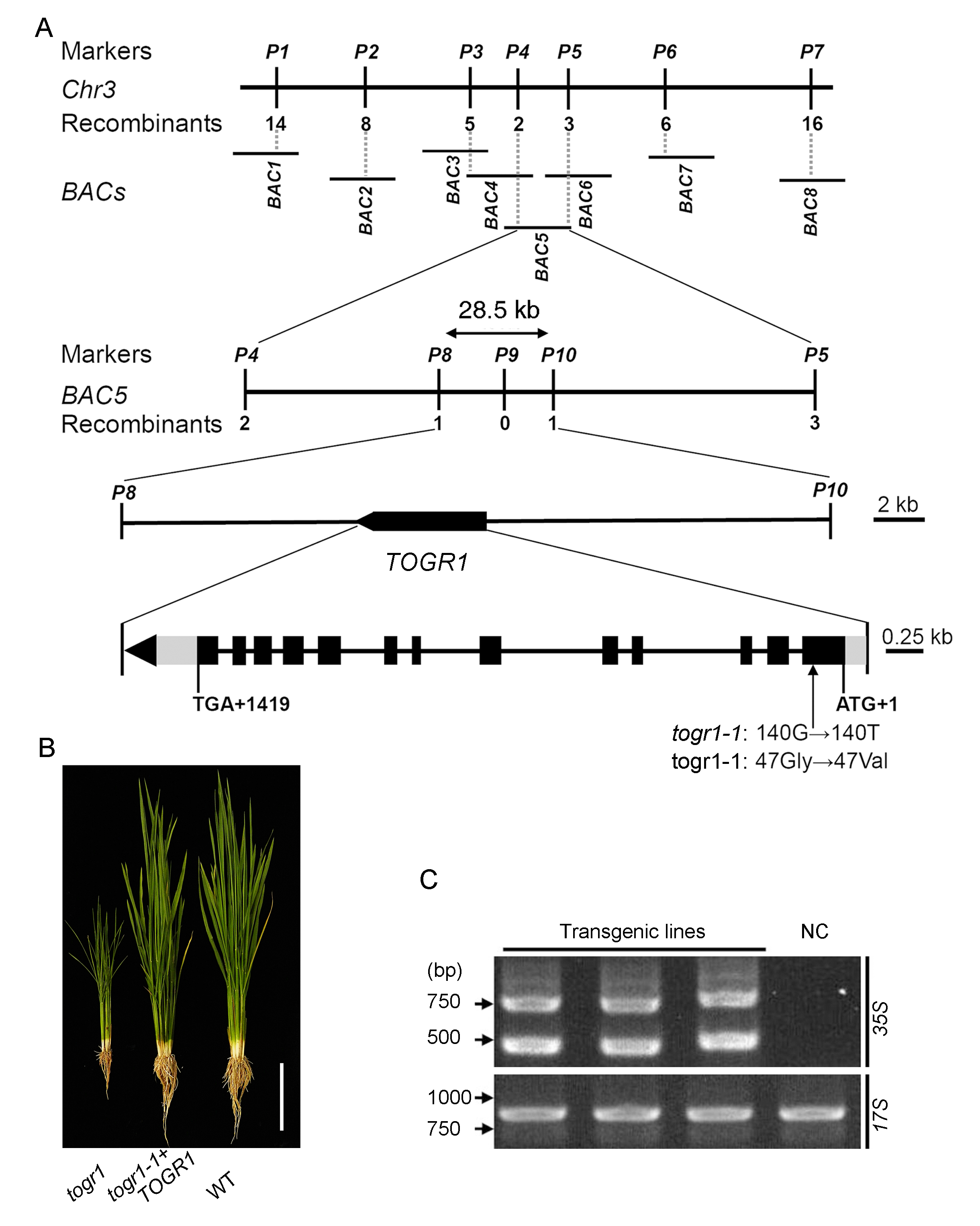

Supplement: S4 Fig — (A) Fine mapping and cloning of the TOGR1 locus. The mutation site at the togr1-1 genome is indicated by an inverted vertical arrow. P1 to P10 are polymorphic DNA markers developed in this work. Numbers of recombinants are shown under each marker. BAC1-BAC8 correspond to AC151537, AC139174, AC135792, AC145388, AC133930, AC146718, AC105747 and AC093018, respectively. (B) Growth of the complemented togr1-1+TOGR1 plant in comparison that of WT and togr1 in Beijing’s summer-autumn field. A construct containing TOGR1pro::TOGR1 was used for complementation transformation. Scale bar: 15 cm. (C) PCR identification of TOGR1 complementation transgenic lines. Primers 35SF and 35SR were used to confirm the presence of the transgene. Partial 17S rRNA gene amplified by primers 17SF/17SR was used as reference. NC, negative control of non-transgenic plant. Sizes of DNA markers are given on the left. (TIF) [file pgen.1005844.s004.tif]

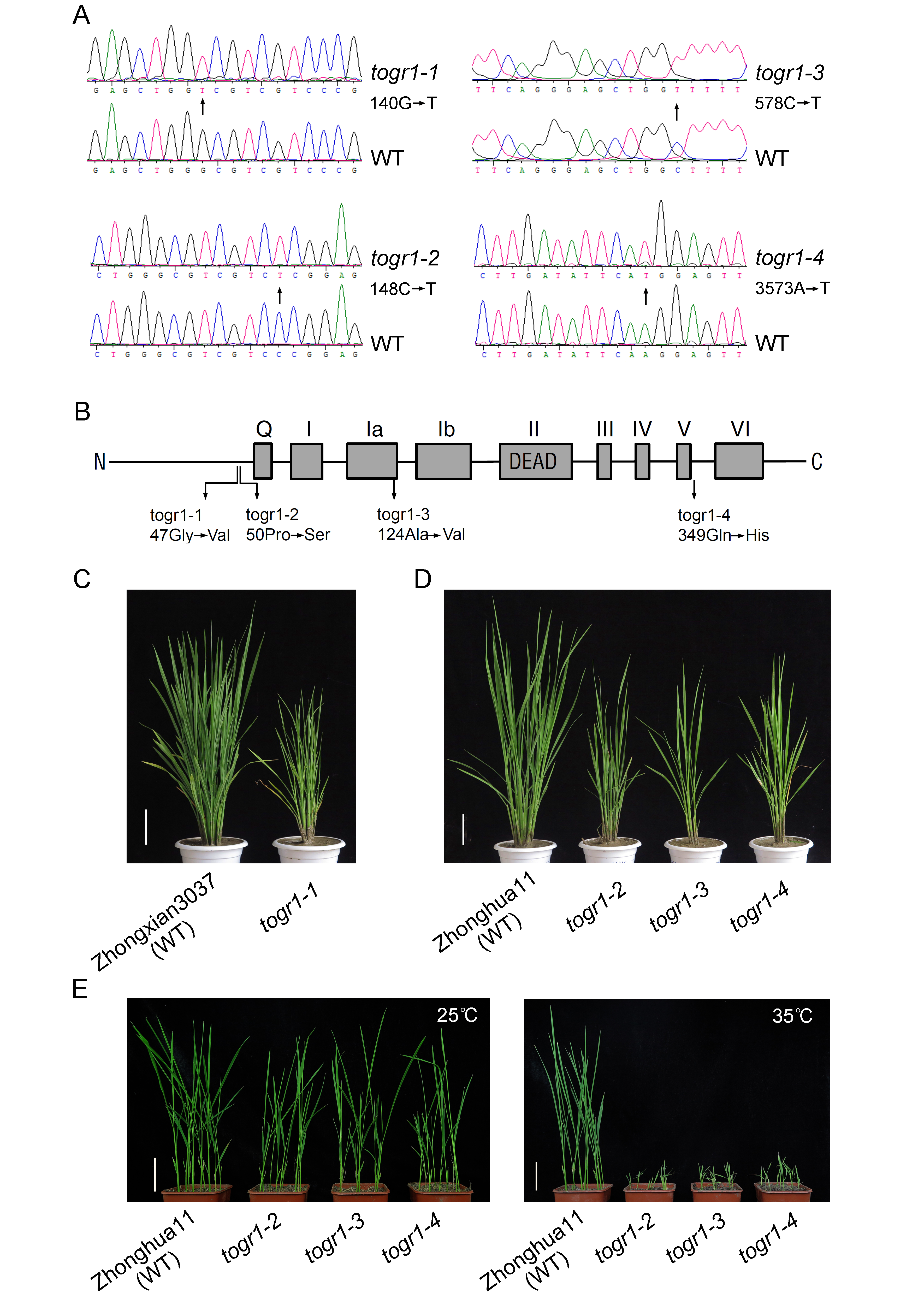

Supplement: S5 Fig — (A and B) Mutation sites of four allelic mutants of togr1. togr1-1 is in Zhongxian 3037 background, and togr1-2 to -4 are in Zhonghua 11 background. (C and D) Three-month-old plants grown in a paddy field under Beijing’s summer-autumn conditions. Scale bars: 10 cm. (E) Two-week-old seedlings grown in chamber at 25 and 35°C. Scale bars: 5 cm. (TIF) [file pgen.1005844.s005.tif]

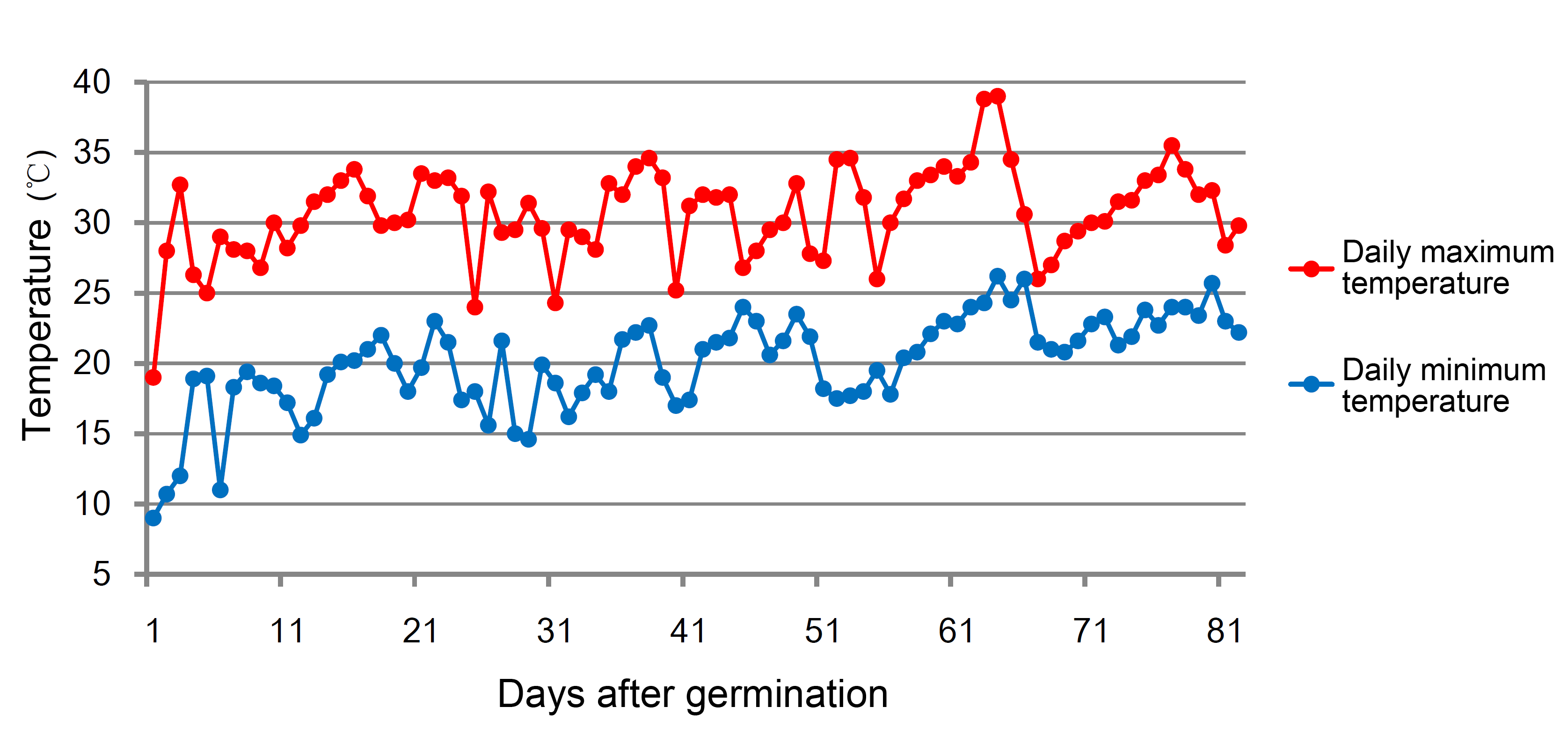

Supplement: S6 Fig — (TIF) [file pgen.1005844.s006.tif]

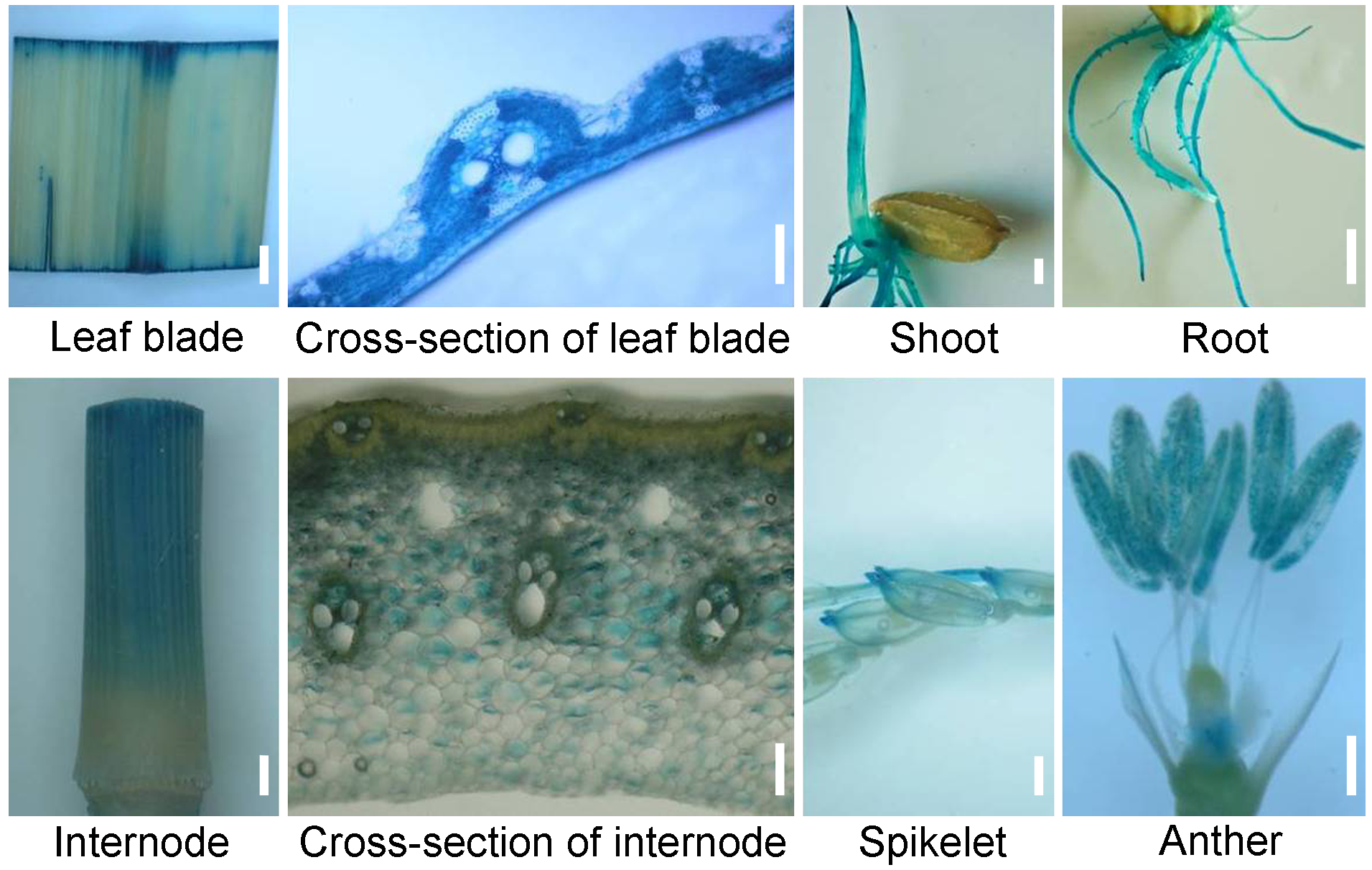

Supplement: S7 Fig — β-Glucuronidase (GUS) histochemical analysis of TOGR1 expression shows its expression in leaf blade, seedling shoot, roots, internode, and anthers. Plants were transformed with a construct containing TOGR1pro::GUS. Scale bars: leaf blade, 2 mm; cross-section of leaf blade and internode, 0.1 mm; seedling shoot, roots and spikelet, 5 mm; internode and anther, 1 mm. (TIF) [file pgen.1005844.s007.tif]

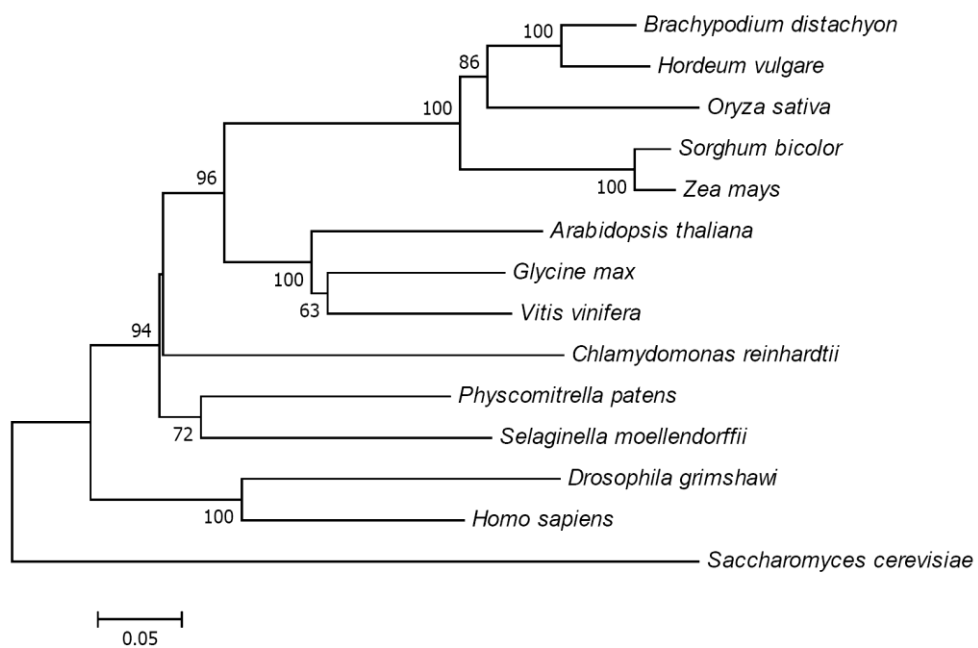

Supplement: S9 Fig — The tree was constructed using the neighbor-joining method based on a Clustal W alignment. Bootstrap values based on 1000 replications are indicated in their respective nodes. The scale bar indicates genetic distance based on branch length. (PDF) [file pgen.1005844.s009.pdf]

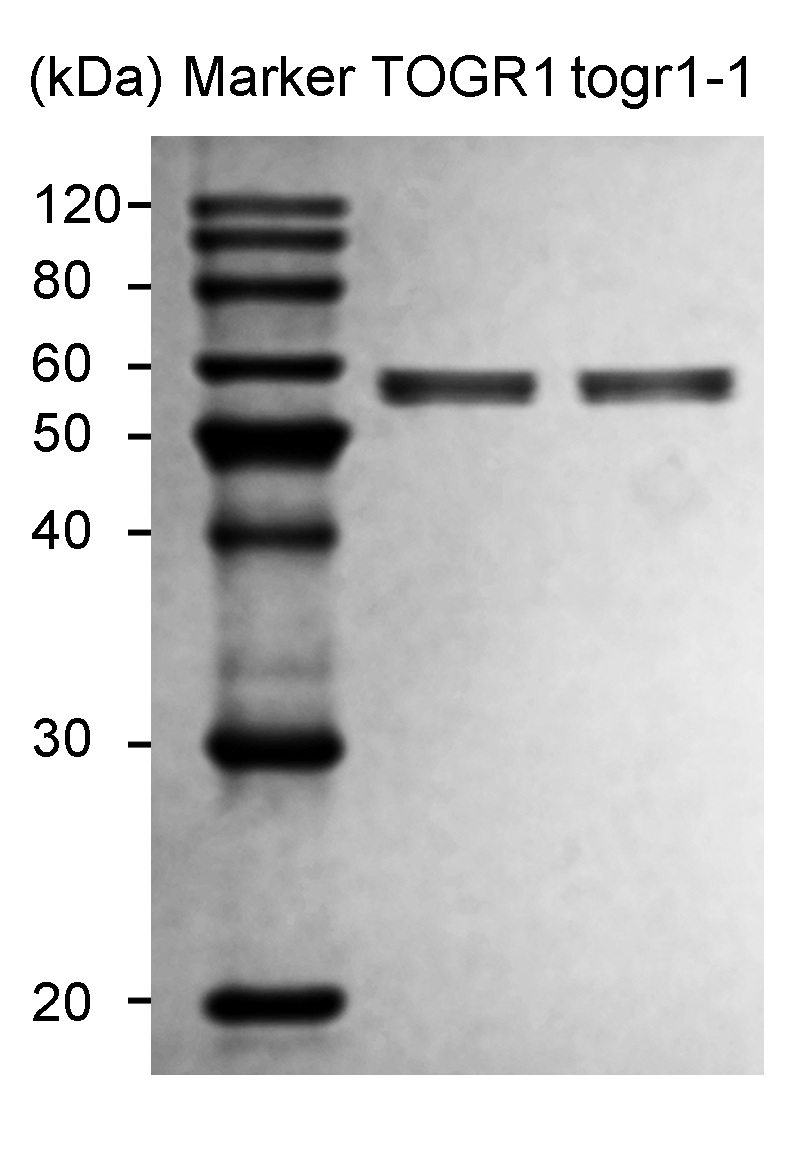

Supplement: S10 Fig — Desired proteins were cleaved from N-His-SUMO-fusion proteins purified from E. coli lysates and the GST-tagged protease used for cleavage was removed by using GST binding resin. Proteins were run on 12% polyacrylamide gel. (TIF) [file pgen.1005844.s010.tif]

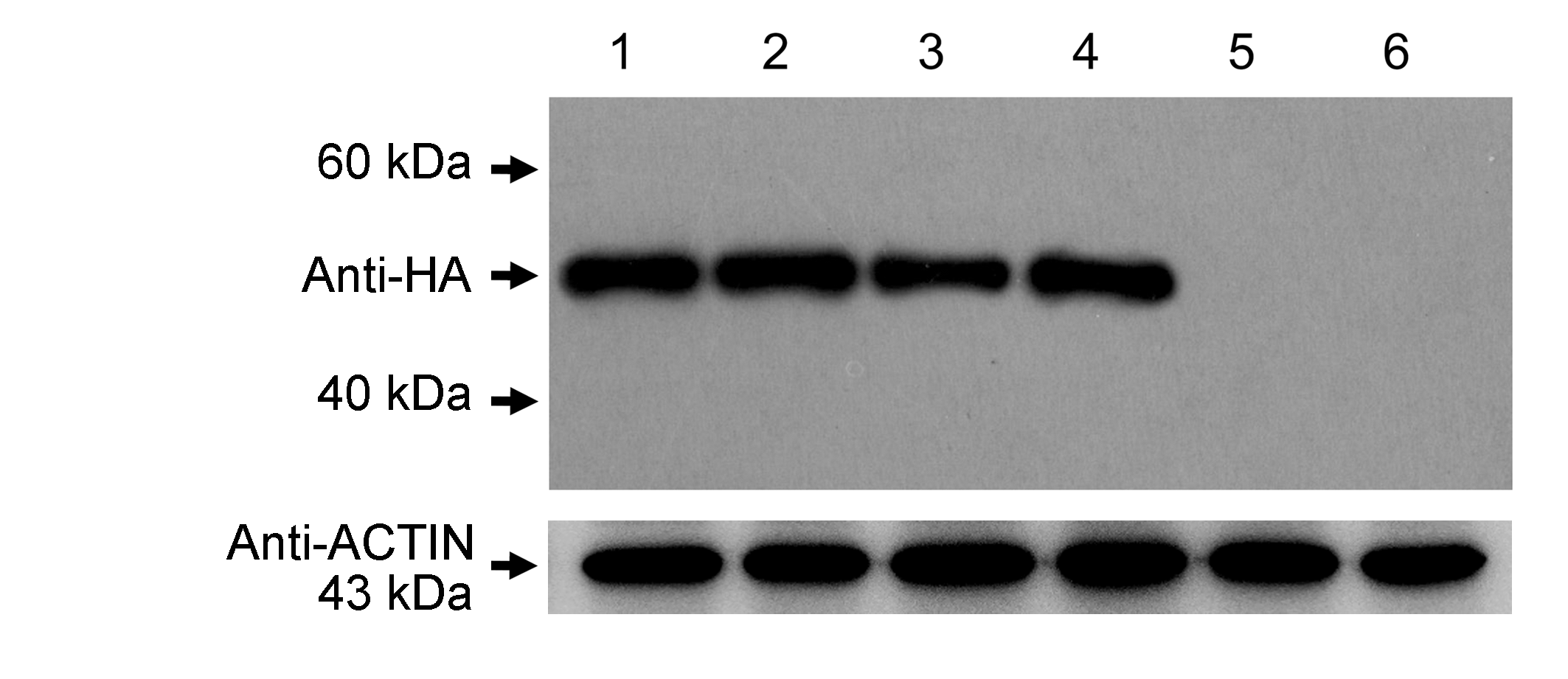

Supplement: S12 Fig — Lanes 1 and 2, tetO7::Rrp3 yeast strain carrying GAL1::TOGR1:HA and GAL1::togr1:HA, respectively; lanes 3 and 4, togr1 rice plants carrying UBi-1::TOGR1:HA. Yeast carrying empty pYES2 (lane 5) and wild-type rice (lane 6) were used as negative controls. Total protein was extracted from yeast or rice leaf blades and run on 12% polyacrylamide gel. HA-tagged proteins were detected using mouse anti-HA monoclonal antibodies. ACTIN was analyzed by anti-ACTIN as an internal control. (TIF) [file pgen.1005844.s012.tif]

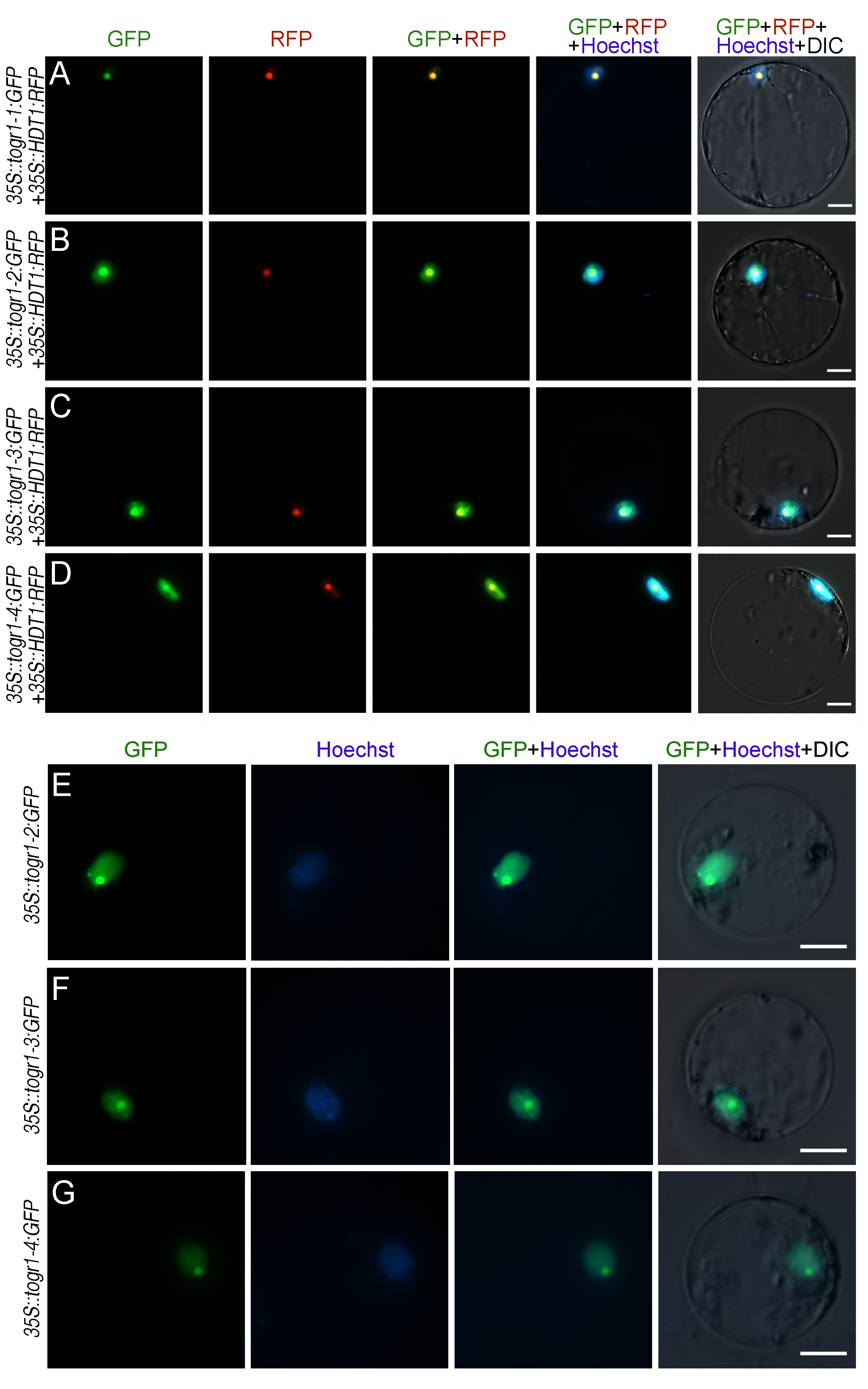

Supplement: S13 Fig — Subcellular localization of togr1-1-GFP (A, green), togr1-2-GFP (B and E, green), togr1-3-GFP (C and F, green) and togr1-4-GFP (D and G, green) were visualized and photographed in protoplasts. The nucleus was stained by Hoechst dye (purple). HDT1-RFP (red) was used as a nucleolus marker. Protoplasts were prepared from rice seedlings and were transformed with respective constructs. Scale bars: 10 μm. (TIF) [file pgen.1005844.s013.tif]

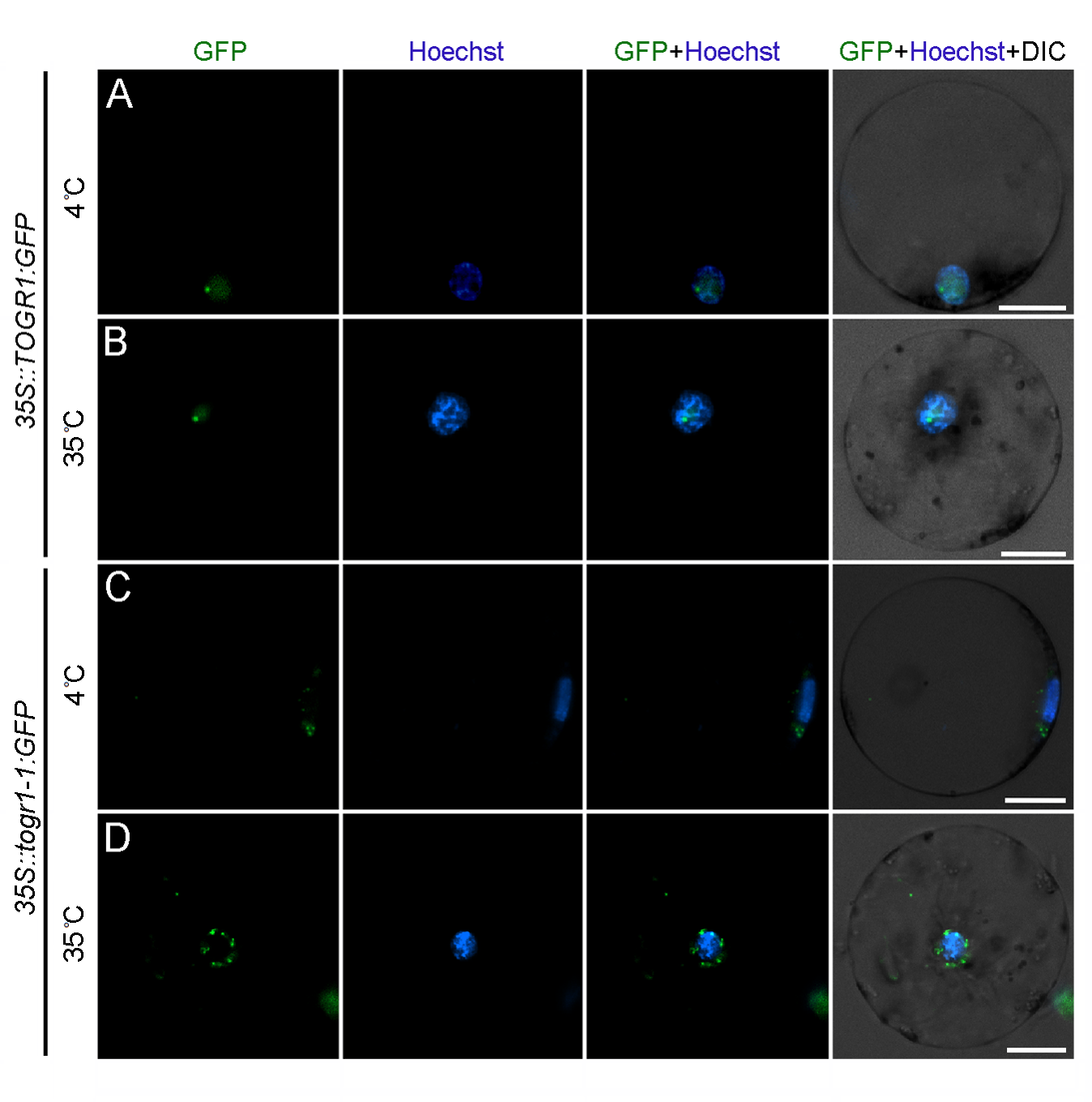

Supplement: S14 Fig — Subcellular localization of TOGR1-GFP (A and B, green) and togr1-1-GFP (C and D, green) were visualized and photographed in protoplasts. The nucleus was stained by Hoechst dye (purple). Protoplasts were prepared from rice seedlings and were transformed with constructs containing 35S::TOGR1:GFP (A and B) and 35S::togr1-1:GFP (C and D), respectively. Transformed protoplasts were either incubated at 4°C for one day (A and C) or treated with 22h°C 20+ 2h 35°C (B and D). Scale bars: 10 μm. (TIF) [file pgen.1005844.s014.tif]

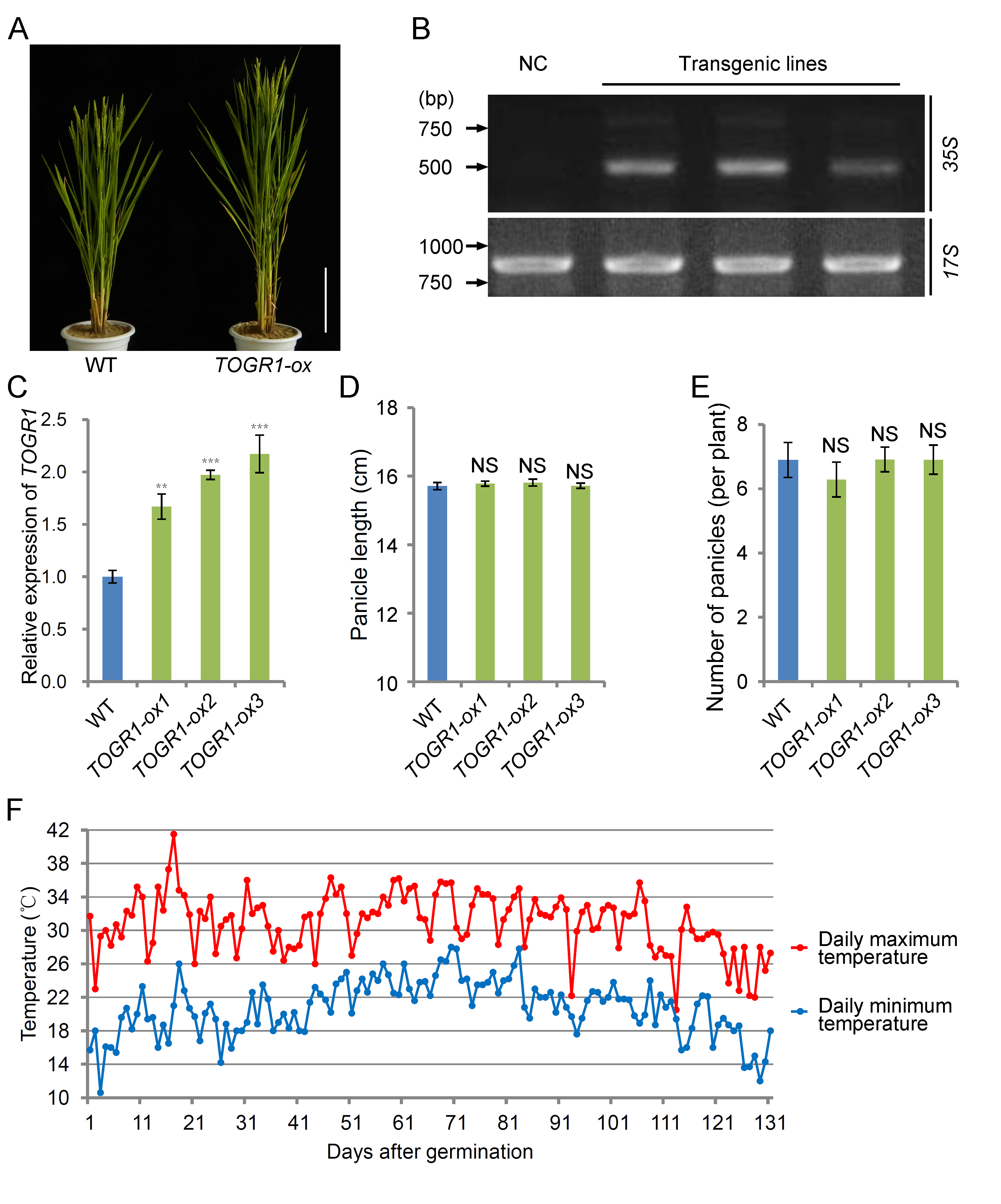

Supplement: S15 Fig — (A) WT and TOGR1-overexpressing plants at heading stage. Scale bar: 20 cm. (B) PCR identification of TOGR1-overexpressing transgenic lines. Primers 35SF and 35SR were used to confirm the presence of the transgene. Partial 17S rRNA gene amplified by primers 17SF/17SR was used as reference. NC, negative control of non-transgenic plant. Sizes of DNA markers are given on the left. (C) Transcript level of TOGR1 in the WT and TOGR1-overexpressing transgenic lines. The average transcript level of TOGR1 in WT is set to 1. (D and E) Comparison of panicle length and number of panicles per plant between the WT and TOGR1-overexpressing transgenic lines grown in Beijing’s summer-autumn fields. Data are represented as mean ± SEM (C, n = 3 replicates; D, n = 30 plants; E, n = 120 panicles). Asterisks indicate statistical significance compared to WT: NS, not significant; **p < 0.01; ***p < 0.001; one-way ANOVA with a priori contrasts. (F) Daily maximum and minimum temperatures of the rice-growing fields in Beijing from May 13th to Sep 20th (summer-autumn) in 2014. (TIF) [file pgen.1005844.s015.tif]

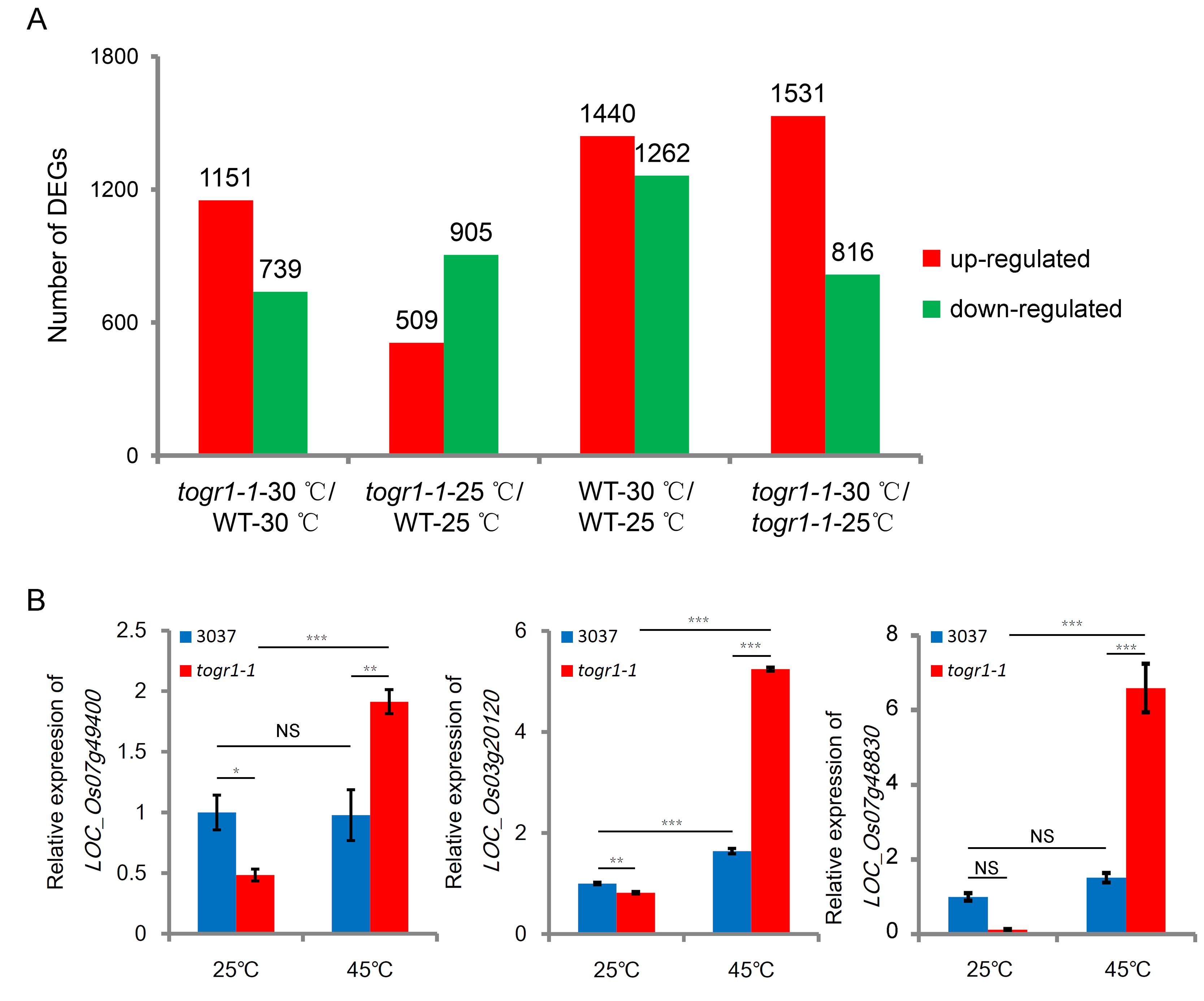

Supplement: S16 Fig — (A) Wild-type and togr1 seedlings were grown at 25°C (WT-25°C and togr1-25°C) and 30°C (WT-30°C and togr1-30°C) for 18 days, respectively. Total RNA was extracted from leaf blades. Transcriptome data were obtained from RNA sequencing. Numbers of DEGs between samples are compared as indicated. (B) Transcript levels of LOC_Os07g49400, LOC_Os03g20120 and LOC_Os07g48830 in the WT and togr1-1 seedlings after one-day of 45°C heat stress treatment. Seedlings grown at 25°C were used as controls. The average transcript levels of the three genes in WT at 25°C are set to 1. Data are represented as mean ± SEM (n = 3 plants). Asterisks indicate statistical significance: NS, not significant; *p < 0.05; **p < 0.01; ***p < 0.001; one-way ANOVA with a priori contrasts. (TIF) [file pgen.1005844.s016.tif]
