# Supplementary material for: Nucleolar DEAD-Box RNA Helicase TOGR1 Regulates Thermotolerant Growth as a Pre-rRNA Chaperone in Rice
Source: PLoS Genet. 2016 Feb 5;12(2):e1005844. doi: 10.1371/journal.pgen.1005844 (PMC4743921; doi:10.1371/journal.pgen.1005844)
Supplement: S8 Fig — Amino acid sequences were aligned using Clustal W. Alignment was shaded using GenDoc. Identical amino acid residues and conservative changes are indicated in black and grey background, respectively. Nine conserved motifs are labeled. The mutation site in togr1 is marked with asterisk. (PDF) [file pgen.1005844.s008.pdf]

```
                                                                          Q-motif
                                                 *
O. sativa           22 : APAADGGEEQEAEP-PARRPSTFAELGVVPELVAACDAMGWKEPTRIQAEAIPHALEGRD
```

```
O. sativa            1 : ---------------------------------------MAKKKDVEVEELDEEVVAAAA
H. vulgare           1 : -------------------------------------------------------MAVVD
B. distachyon        1 : -------------------------------------------------------MAVE-
Z. mays              1 : --------------------------------------------------------MANG
S. bicolour          1 : --------------------------------------------------------MANG
A. thaliana          - : ------------------------------------------------------------
V. vinifera          1 : --------------------------------------MSATGGAAGGQEEDKKPTDQGA
G. max               - : ------------------------------------------------------------
P. patens            - : ------------------------------------------------------------
S. moellendorffii    - : ------------------------------------------------------------
S. cerevisiae        1 : MSKIVKRKEKKANDELTSLAEKIRAKALENQKKLIEAEKEGGSESDSEEDATAEKKKVLK
H. sapiens           1 : ---------------------------------------------------MAAPEEHDS
D. grimshawi         1 : ------------------------MSETSEDEQAQLQTSEEELSDEEGQEENAEDVDEDN
C. reinhardtii       - : ------------------------------------------------------------

                                                                   Q-motif
                                                  *
O. sativa           22 : APAADGGEEQEAEP-PARRPSTFAELGVVPELVAACDAMGWKEPTRIQAEAIPHALEGRD
H. vulgare           6 : KAAAGGGEAQQREPEPAAEASTFAELGICRELVEACDAMGWKQPTKIQAGAIPHALQGRD
B. distachyon        5 : -------REPELEPEPVAEASTFAELGICKELVDACDSMGWKEPTKIQAGAIPHALQGRD
Z. mays              5 : DAAVEVGGNGEQEX-AARQASTFAELGICAELVEACDAMGWKEPTRIQAEAIPHALQGKD
S. bicolour          5 : DAAVEAGGNGKEEP-AARQASTFAELGICAELVEACDAMGWKEPTRIQAEAIPHALQGKD
A. thaliana          1 : ------------MEEENEVVKTFAELGVREELVKACERLGWKNPSKIQAEALPFALEGKD
V. vinifera         23 : HINLKVKGQIFRMAEDNKEVKSFKDLGICEQLVEACENLGWKTPSKIQAEAIPHALEGKD
G. max               1 : ------------MGEENEETKTFKDLGLSESLVEACEKLGWKNPLKIQTEAIPLALEGKD
P. patens            1 : --MGSAAAAAEDSSAEEKKAPTFRSLGVCEQLAEACDALGWKAPTQIQVDAVPVALKGHD
S. moellendorffii    1 : --MAAAAA-----------VPSFGELGVREELVDACTKLGWKAPTPIQVEALPLALQGRD
S. cerevisiae       61 : SKSKSTVSTQNENTNEDESFESFSELNLVPELIQACKNLNYSKPTPIQSKAIPPALEGHD
H. sapiens          10 : PTEASQPIVEEEET------KTFKDLGVTDVLCEACDQLGWTKPTKIQIEAIPLALQGRD
D. grimshawi        37 : VDDQSATSSDEEETPKAEQPLTWKDLGLSETLCKACDELKWKAPSKIQKEAIPVALQGKD
C. reinhardtii       1 : ----MGGPDKLTDAERAAAIAAFEKLGICTQLAEAAAGLGWKAPSHIQEQAIPHLLAGQD

                              I                                      Ia
O. sativa           81 : LIGLGQTGSGKTGAFALPIIQALLKQDKPQ---------A-LFACVLSPTRELAFQIGQQ
H. vulgare          66 : VIGLGQTGSGKTGAFALPIIQALLEHRQP------------FFACVMSPTRELAIQIAEQ
B. distachyon       58 : LIGLGQTGSGKTGAFALPIIQALLEHRQP------------FFACVMSPTRELAIQIAEQ
Z. mays             64 : LIALAQTGSGKTGAFALPILQELLSNRQAE---------QSFFACVLSPTRELAIQIAEQ
S. bicolour         64 : LIALAQTGSGKTGAFALPILQELLSNRQAE---------QSFFACVLSPTRELAIQIAEQ
A. thaliana         49 : VIGLAQTGSGKTGAFAIPILQALLEYVYDSEPKKGRRPDPAFFACVLSPTRELAIQIAEQ
V. vinifera         83 : LIGLAQTGSGKTGAFALPILQALLDTP------------QVLFACVLSPTRELAIQIAEQ
G. max              49 : VIGLAQTGSGKTGAFALPILHALLEAPRP----------KDFFACVLSPTRELAIQIAEQ
P. patens           59 : IIGLAQTGSGKTATFALPILQALLENPQP------------LFACVLSPTRELAIQIAEQ
S. moellendorffii   48 : LIGLAQTGSGKTAAFALPILQALFQQCHP------------FFACVLSPTRELAIQISEQ
S. cerevisiae      121 : IIGLAQTGSGKTAAFAIPILNRLWHDQEP------------YYACILAPTRELAQQIKET
H. sapiens          64 : IIGLAETGSGKTGAFALPILNALLETPQR------------LFALVLTPTRELAFQISEQ
D. grimshawi        97 : VIGLAETGSGKTGAFALPILHALLENPQR------------YFALVLTPTRELAFQIGEQ
C. reinhardtii      57 : VIGLAQTGSGKTGAFSLPILQALMDKPQE------------HFALILSPTRELAIQIAEQ

                                                                Ib
O. sativa          131 : FEALGSAIGLSCTVLVGGVDRVQQAVSLAKRPHIVVGTPGRLLDHLTDTKGFSLNKLKYL
H. vulgare         114 : FEALGSGIGLVCSVLVGGVDRMQQVLSIAKRPHIVVGTPGRLLDHLKDTKGFSLTKVKYL
B. distachyon      106 : FEALGSAIGLVCSVLVGGVDRMQQVLSIAKRPHIVVGTPGRLLDHLKDTKGFSLNKVKYL
Z. mays            115 : FEALGSAIGLRCSVLVGGVDRVQQVLSIGKRPHIVVGTPGRLLDHLTETKGFSLKKIKYL
S. bicolour        115 : FEALGSAIGLRCSVLVGGVDRVQQVLSIGKRPHIVVGTPGRLLDHLTETKGFSLKKIKYL
A. thaliana        109 : FEALGADISLRCAVLVGGIDRMQQTIALGKRPHVIVATPGRLWDHMSDTKGFSLKSLKYL
V. vinifera        131 : FEALGSGIGLKCAVLVGGVDHTQQAIALAKRPHIVVGTPGRLMDHLSNTKGFSLRTMKYL
G. max              99 : FEALGSEIGVKCAVLVGGIDMVQQSIKIAKQPHIIVGTPGRVIDHLKHTKGFSLSRLKYL
P. patens          107 : FEALGSGIGLKCAVLVGGIDMMAQSVALAKRPHVVVGTPGRLVDHLTNTKGFSLRTIKYL
S. moellendorffii   96 : FEALGSQIGVRSVAIVGGVSMVDQAVALGKNPHIVVATPGRLLDHLTNTKGFSLRNVKYL
S. cerevisiae      169 : FDSLGSLMGVRSTCIVGGMNMMDQARDLMRKPHIIIATPGRLMDHLENTKGFSLRKLKFL
H. sapiens         112 : FEALGSSIGVQSAVIVGGIDSMSQSLALAKKPHIIIATPGRLIDHLENTKGFNLRALKYL
D. grimshawi       145 : FEALGSGIGIKCCVIVGGMDMVAQGLQLAKKPHIIIATPGRLVDHLENLKGFNLKAIKYL
C. reinhardtii     105 : VEALGSGIGVKSCVLVGGIDMMAQAIALAKRPHVLVGTPGRVVDHLSNTKGFSLKQLKHL
```

```
                             II                               III
O. sativa           191 : VLDEADKLLNVEFQKALDDILNVIP-KERRTFLFSATMTNKVSKLQRACLRNPVKVEVAS
H. vulgare          174 : VLDEADKLLNLEFKESLDDILKEIP-KERRTYLFSATMTKKVSKLQRACLRNPVKVEVSS
B. distachyon       166 : VLDEADKLLNLEFKESLDDILKAIP-KERRTYLFSATMTKKVAKLQRACLRNPVKVEVSS
Z. mays             175 : VLDEADKLLNVEFEKSLDDILSEMP-KDRRTFLFSATMTKKVNKLQRACLRNPAKVEVSS
S. bicolour         175 : VLDEADKLLNVEFEKSLDDILREIP-KDRRTFLFSATMTKKVNKLQRACLRNPAKVEAAS
A. thaliana         169 : VLDEADRLLNEDFEKSLNQILEEIP-LERKTFLFSATMTKKVRKLQRACLRNPVKIEAAS
V. vinifera         191 : VLDEADRLLNDDFEKAIDEILSVIP-RERKTYLFSATMTKKVRKLQRACLRNPVKIEVAS
G. max              159 : VLDEADRLLNEDFEESLNEILQMIP-RERRTFLFSATMTKKVQKLQRVCLRNPVKIEASS
P. patens           167 : VLDEADRLLNMDFEQEIDEILKVIP-KERRTYLFSATMTTKVAKLQRACLKNPVKVEVSA
S. moellendorffii   156 : VLDEADKILHNDFEKEVDEILKVVP-RERKTFLFSATMTNKVAKLQRACLRNPTKVEVSS
S. cerevisiae       229 : VMDEADRLLDMEFGPVLDRILKIIPTQERTTYLFSATMTSKIDKLQRASLTNPVKCAVSN
H. sapiens          172 : VMDEADRILNMDFETEVDKILKVIP-RDRKTFLFSATMTKKVQKLQRAALKNPVKCAVSS
D. grimshawi        205 : VMDEADRILNMDFEVELDKILKVLP-RERRTFLFSATMTKKVKKLQRASLKDPVKVEVSN
C. reinhardtii      165 : VLDEADKLLDMDFEQEIDQILKVIP-RERRTQLFSATMTNKVQKLQRACLDKPVKIEVAH
```

```
                                                                IV
O. sativa           250 : KYSTVDTLRQEFYFVPADYKDCFLVHVLNELPGSMIMIFVRTCESTRLLALTLRNLRFKA
H. vulgare          233 : KYSTVDTLKQEWYFVPADYKDCYLVHVLNELQGSMIMIFVRTCESTRLLALTLRNLGFKA
B. distachyon       225 : KYSTVDTLKQEWYFVPAAYKDCYLVHALNELPGSMIMIFVRTCESTRLLALTLRNLGFKA
Z. mays             234 : KYSTVDSLKQEFYFVPADDKDCYLLHVLNERLESMIMIFVRTCESTRLLALMLRNLGLKA
S. bicolour         234 : KYSTVDSLKQEFYFVPADDKDCYLLHVLNERQDSMIMIFVRTCESTRLLALMLRNLGLKA
A. thaliana         228 : KYSTVDTLKQQYRFVAAKYKDCYLVYILSEMPESTSMIFTRTCDGTRFLALVLRSLGFRA
V. vinifera         250 : KYSTVDTLKQQYRFVPAKYKECYLVYILTELSGSTTMVFTRTCDATRLLALLLRNLGLRA
G. max              218 : KYSTVDTLKQQYRFLPAKHKDCYLVYILTEMAGSTSMVFTRTCDATRLLALILRNLGLKA
P. patens           226 : KYSTVDSLKQEYLFIPAKYKDCYLVYILNELAGNTAMVFTRTCEATRKLSLVLRNLGFVA
S. moellendorffii   215 : KYTTALTLKQEYVFVPAKHKDCYFVFLLNEMAGSTAMVFTRTCDSTRRLALILRSLGFGA
S. cerevisiae       289 : KYQTVDTLVQTLMVVPGGLKNTYLIYLLNEFIGKTMIIFTRTKANAERLSGLCNLLEFSA
H. sapiens          231 : KYQTVEKLQQYYIFIPSKFKDTYLVYILNELAGNSFMIFCSTCNNTQRTALLLRNLGFTA
D. grimshawi        264 : KYQTVDQLQQYYLFIPVKYKDVYLVHILNELAGNSFMIFCSTCNNTVKTALMLRALGLAA
C. reinhardtii      224 : KYSTVDTLRQQYVFIPAKYKDCYLAYVINELSGSTFMIFTRTCESTRRIALLLRNLGFGA
```

```
                                                           V
O. sativa           310 : ISISGQMSQDKRLGALNRFKTKDCNILICTDVASRGLDIQGVDVVINYDIPMNSKDYVHR
H. vulgare          293 : LSISGQMSQDKRLGALNKFKAKDFNILICTDVASRGLDIQGVDVVMNYDIPMNSKDYVHR
B. distachyon       285 : LSISGQMSQDKRLGALNKFKAKDFNILICTDVASRGLDIQGVDVVINYDIPMNSKDYVHR
Z. mays             294 : MSISGQMSQDKRLGALNRFKAKDCNILICTDVASRGLDIQGVDMVINYDIPINSKDYVHR
S. bicolour         294 : MSISGQMSQDKRLGALNRFKAKDCNILICTDVASRGLDIQGVDMVINYDIPMNSKDYVHR
A. thaliana         288 : IPISGQMTQSKRLGALNKFKAGECNILVCTDVASRGLDIPSVDVVINYDIPTNSKDYIHR
V. vinifera         310 : IPISGHMSQAKRLGALNKFKAGECNILICTDVASRGLDIPSVDMVINYDIPSNSKDYIHR
G. max              278 : IPINGHMSQSKRLGALNKFKSGECNILLCTDVASRGLDIPTVDMVINYDIPTNSKDYIHR
P. patens           286 : IPISGQMSQPKRLGALAKFKAGDCNILICTDVASRGLDIPSVNLVINYDIPTNSKDYIHR
S. moellendorffii   275 : IPISGQMSQSKRSGALLKFKAGDRNLLICTDVASRGLDIPSVDVVINYDIPTNSKDYIHR
S. cerevisiae       349 : TALHGDLNQNQRMGSLDLFKAGKRSILVATDVAARGLDIPSVDIVVNYDIPVDSKSYIHR
H. sapiens          291 : IPLHGQMSQSKRLGSLNKFKAKESSILLATDVASRGLDIPHVDVVVNFDIPTHSKDYIHR
D. grimshawi        324 : IPLHGQMSQNKRLAALNKFKAKNRSILISTDVASRGLDIPHVDVVVNFDIPTHSKDYIHR
C. reinhardtii      284 : VPIHGHMSQPKRLGALNKFKSGERNILVATDVASRGLDIPSVDVVINFDVPQNSKDYVHR
```

```
                            VI
O. sativa           370 : VGRTARAGNTGYAVSLVNQYEAMWFKMIEKLLGYEIPDRKVDNAEIMILRERISDSKRIA
H. vulgare          353 : VGRTARAGQSGYAVSFVNQYEAEWFKLIEQLLGRQIPDRKVDMDEILILREHISDSKRIA
B. distachyon       345 : VGRTARAGKSGYAVSLVNQYEGQWFKMIEALLGKEIDLRKVDSDEIMILREHISDSRRIA
Z. mays             354 : VGRTARAGRSGYAVSLVNQYEAQWFVLIEQLLGKKIDQCKVDRDEVLILKGPISDAKRIA
S. bicolour         354 : VGRTARAGRSGYAVSLVNQYEAQWFVLIEKLLGKQIDQRKVDRDEVMILKGPISDAKRIA
A. thaliana         348 : VGRTARAGRSGVGISLVNQYELEWYIQIEKLIGKKLPEYPAEEDEVLSLLERVAEAKKLS
V. vinifera         370 : VGRTARAGRSGVAISLVNQYELEWYIQIEKLIGKKLPEFPAQEEEVLLLLERVTEAKRIS
G. max              338 : VGRTARAGRSGVAISLVNQYELEWYIQIEKLIGKKLPEYPAQEEEVLLLEERVGEAKRLA
P. patens           346 : VGRTARAGRSGRAISMVCQYDVELYQKIEELIGKKLPEFSHQQEEVLLMLERVSEAQRLA
S. moellendorffii   335 : VGRTARAGRSGRAISIVTQYDVDLYKRIEDLIGMTLPEFPAKADEVLLLEERVSEAQRLA
S. cerevisiae       409 : VGRTARAGRSGKSISLVSQYDLELILRIEEVLGKKLPKESVDKNIILTLRDSVDKANGEV
H. sapiens          351 : VGRTARAGRSGKAITFVTQYDVELFQRIEHLIGKKLPGFPTQDDEVMMLTERVAEAQRFA
D. grimshawi        384 : VGRTARAGRSGQAITIVSQYDIELYQRIEQLLGKQLPLYKCEEDEVMALQERVAEAQRTA
C. reinhardtii      344 : VGRTARAGRSGRSVTIVTQYDVELFQKIEHLTGVKMEQFQAERREEVLLLLERVSDAQRLA
```

```
O. sativa           430 : LTTMKEGGGHKK------KRRKNEDDEE-------EEERNAPVSR---------------
H. vulgare          413 : LTKLKEDGGHKK------RRKAAEDEDD-------EEEEAPRGHRQ--------------
B. distachyon       405 : LTKLKEDGGHKK------RRKQDDDDDE-------EEEKAPRGHR---------------
Z. mays             414 : LTKLKDSGGHKK------RRNVGDDDEE-------VEDHSSHSRR---------------
S. bicolour         414 : LTKLKDSGGHKK------RRKAGDDDEE-------VEDYS-HSKR---------------
A. thaliana         408 : AMNMKESGGRK-------RRGEDDEESERFLGGNKDRGNKERGGNK--------------
V. vinifera         430 : QMKVKETGGKKK------RRGGDEGE----------EEIDRYLASK--------------
G. max              398 : VMKMKETGGKKK------RRGEEDYDG---------EDIDKYLGLK--------------
P. patens           406 : TMHMREKDGAKKGK----RRGHGDDDGD-------DNRGPGRPEKG--------------
S. moellendorffii   395 : AMHIREKDASKKSK----KRRNGDDDEA-------DDGG----YMA--------------
S. cerevisiae       469 : VMEMNRRNKEKI------ARGKGRRGRM--------------------------------
H. sapiens          411 : RMELREHGEKK------KRSREDAGDN--------DDTEGAIGVRN-----------KVA
D. grimshawi        444 : KLEFKDLEDSKGYKGGRGNKRTAASDNQ-------DDSEQFTGARKRMKPMGGRGGGKGA
C. reinhardtii      404 : TMQMKEADAGKKG-----KRKADDDDEG-----------AGGGGRG--------------

O. sativa           462 : -------KSKSFNKSRRR----
H. vulgare          446 : -------KSRSFKKSSRR----
B. distachyon       437 : -------KPKSFKKSSRR----
Z. mays             446 : --------PKSLKRSNRR----
S. bicolour         445 : --------PKSFKKSNRR----
A. thaliana         447 : -------DKKSSKKFKR-----
V. vinifera         460 : -------NGKSSKKLKKR----
G. max              429 : -------EGKSSKKFRRR----
P. patens           441 : -------GTRPKKQFKKSR---
S. moellendorffii   426 : -------GKR--KQSKKR----
S. cerevisiae       491 : -------MTRENMDMGER----
H. sapiens          446 : GGK-------MKKRKGR-----
D. grimshawi        497 : GGRGLGRGGAAKKNWGRGKNKN
C. reinhardtii      434 : -------GPKPKLGGGRRKY--
```
