# Supplementary material for: Nucleolar DEAD-Box RNA Helicase TOGR1 Regulates Thermotolerant Growth as a Pre-rRNA Chaperone in Rice
Source: PLoS Genet. 2016 Feb 5;12(2):e1005844. doi: 10.1371/journal.pgen.1005844 (PMC4743921; doi:10.1371/journal.pgen.1005844)
Supplement: S11 Fig — Sequence of the 17S rRNA region is shown in bold letters. (PDF) [file pgen.1005844.s011.pdf]

TCCCACGCGTGGTGGCCCTCCGGTAGGCCGTCCTCCCAGCAGACCAGCCGTGCTCCGCGCGGCAGGATGCTTGGGCGGCCTTG

CCGCCGTGGCTGCGTAGCGTATGAGCAGCTTTGGACCGGTGTATGCTCGCAGGACCCCCGCCCTCGTGCGGCCGACTGCCGGC

TCCCGGGCCCCGTCACTCCACGGCCGTCCACGCGCCGTGCCGCCCCAGGCTTCAAGAGATGCTTGCGCGCTGCTACCCGTCCC

ACGGGCAGAGGTGCTCGCACACGTCCGCCGCGCCGCGGGCGCCCCACCGGGCGTCCCGCGGCGGCTCGACGGCGCGAGCGGCG

TGGCCTCGCGGCGCCCGGCACCCAAGCGTGCCGGCGCTGCCAAGGCCACCTCGCGCGTGCCATTGGTCCCGGATGCCGCCCAC

GATACAGGCTCACGGCGGCCCCGCCCCGTGCCTACCCATAAGCGAGATGCTCTCGGAAGACGACAGCCCGCCCGGCCGCCGCC

GTGTCCGCCGCTCCCGACCCGGGGGCGGCGGCGACGCGCGTCGGACGGCGCGGGCTCGTCGCGGAGGACGTGCT**ACCTGGTT**

GATCCTGCCAGTAGTCATATGCTTGTCTCAAAGATTAAGCCATGCATGTGCAAGTATGAACTAATTCGAACTGTGAAACTGC

GAATGGCTCATTAAATCAGTTATAGTTTGTTTGATGGTACGTGCTACTCGGATAACCGTAGTAATTCTAGAGCTAATACGTG

CAACAAACCCCGACTTCCGGGAGGGGCGCATTTATTAGATAAAAGGCTGACGCGGGCTCCGCCCGCTGATCCGATGATTCAT

GATAACTCGACGGATCGCACGGCCCTCGTGCCGGCGACGCATCATTCAAATTTCTGCCCTATCAACTTTCGATGGTAGGATA

GGGGCCTACCATGGTGGTGACGGGTGACGGAGAATTAGGGTTCGATTCCGGAGAGGGAGCCTGAGAAACGGCTACCACATCC

AAGGAAGGCAGCAGGCGCGCAAATTACCCAATCCTGACACGGGGAGGTAGTGACAATAAATAACAATACCGGGCGCTTTAGT

GTCTGGTAATTGGAATGAGTACAATCTAAATCCCTTAACGAGGATCCATTGGAGGGCAAGTCTGGTGCCAGCAGCCGCGGTA

ATTCCAGCTCCAATAGCGTATATTTAAGTTGTTGCAGTTAAAAAGCTCGTAGTTGGACCTTGGGCCGGGCCGGCCGGTCCGC

CTCACGGCGAGCACCGACCTGCTCGACCCTTCTGCCGGCGATGCGCTCCTGGCCTTAACTGGCCGGGTCGTGCCTCCGGCGC

CGTTACTTTGAAGAAATTAGAGTGCTCAAAGCAAGCCATCGCTCTGGATACATTAGCATGGGATAACATCATAGGATTCCGG

TCCTATTGTGTTGGCCTTCGGGATCGGAGTAATGATTAATAGGGACAGTCGGGGGCATTCGTATTTCATAGTCAGAGGTGAA

ATTCTTGGATTTATGAAAGACGAACAACTGCGAAAGCATTTGCCAAGGATGTTTTCATTAATCAAGAACGAAAGTTGGGGGC

TCGAAGACGATCAGATACCGTCCTAGTCTCAACCATAAACGATGCCGACCAGGGATCGGCGGATGTTGCTTATAGGACTCCG

CCGGCACCTTATGAGAAATCAAAGTCTTTGGGTTCCGGGGGGAGTATGGTCGCAAGGCTGAAACTTAAAGGAATTGACGGAA

GGGCACCACCAGGCGTGGAGCCTGCGGCTTAATTTGACTCAACACGGGGAAACTTACCAGGTCCAGACATAGCAAGGATTGA

CAGACTGAGAGCTCTTTCTTGATTCTATGGGTGGTGGTGCATGGCCGTTCTTAGTTGGTGGAGCGATTTGTCTGGTTAATTC

CGTTAACGAACGAGACCTCAGCCTGCTAACTAGCTATGCGGAGCCATCCCTCCGCAGCTAGCTTCTTAGAGGGACTATGGCC

GTTTAGGCCACGGAAGTTTGAGGCAATAACAGGTCTGTGATGCCCTTAGATGTTCTGGGCCGCACGCGCGCTACACTGATGT

ATCCAACGAGTATATAGCCTTGGCCGACAGGCCCGGGTAATCTTGGGAAATTTCATCGTGATGGGGATAGATCATTGCAATT

GTTGGTCTTCAACGAGGAATGCCTAGTAAGCGCGAGTCATCAGCTCGCGTTGACTACGTCCCTGCCCTTTGTACACACCGCC

CGTCGCTCCTACCGATTGAATGGTCCGGTGAAGTGTTCGGATCGCGGCGACGGGGGCGGTTCGCCGCCCCCGACGTCGCGAG

AAGTCCATTGAACCTTATCATTTAGAGGAAGGAGAAGTCGTAACAAGGTTTCCGTAGGTGAACCTGCGGAAGGATCATTGTC

GTGACCCTGACCAAAACAGACCGCGAACGCGTCACCCCTGCCCGCCGAGCGCTCGCGCGCGAGGCGACCGAGGCCCCCGGGCC

GCAACAGAACCCACGGCGCCGACGGCGTCAAGG
