# Supplementary material for: Nucleolar DEAD-Box RNA Helicase TOGR1 Regulates Thermotolerant Growth as a Pre-rRNA Chaperone in Rice
Source: PLoS Genet. 2016 Feb 5;12(2):e1005844. doi: 10.1371/journal.pgen.1005844 (PMC4743921; doi:10.1371/journal.pgen.1005844)
Supplement: S1 Table — (PDF) [file pgen.1005844.s017.pdf]

S1 Table. List of the oligonucleotides used for northern analysis and circular RT-PCR.

Name	sequence	Note
S1	5′-GTCCTCCGCGACGAGCCCGC-3′	northern blot analysis
S2	5′-TGCTGCCTTCCTTGGATGTG-3′	
S3	5′-GCGGTCTGTTTTGGTCAGGGTCACGA-3′	
S4	5′-GCGCGGAGCGTCTTTTGGC-3′	
S5	5′-GAGGCGTTCGCTCTCGGTGC-3′	
S6	5′-TGGATCTCAGTGGATCGTGG-3′	
CRT1	5′-GTCCTCCGCGACGAGCC-3′	circular RT-PCR
CRTF	5′-TCGTGACCCTGACCAAAACAG-3′	
CRTR	5′-GGGTCGGGAGCGGCGGACAC-3′	
