# Supplementary material for: Nucleolar DEAD-Box RNA Helicase TOGR1 Regulates Thermotolerant Growth as a Pre-rRNA Chaperone in Rice
Source: PLoS Genet. 2016 Feb 5;12(2):e1005844. doi: 10.1371/journal.pgen.1005844 (PMC4743921; doi:10.1371/journal.pgen.1005844)
Supplement: S2 Table — (PDF) [file pgen.1005844.s018.pdf]

S2 Table. Relative transcript level of *TOGR1* in 38 rice accessions.

Name	Original producing area	Mean transcript level of *TOGR1*	S.E.	Mean plant height (cm)	Type
Wuke-4	China	1.00	0.0307	107.5	*indica*
Hongmangnuo	China	1.13	0.0855	153.7	*temperate japonica*
Lijianghei	China	0.72	0.038	165	*temperate japonica*
Maguzhi	China	0.63	0.0261	155	*temperate japonica*
Nipponbare	Japan	0.59	0.1018	99.8	*temperate japonica*
Yandao8	China	0.06	0.0013	67.4	*temperate japonica*
Zhonghua11	China	0.79	0.06	112	*temperate japonica*
O. rufipogon	China	1.14	0.1198	125	*rufipogon*
9311	China	0.76	0.0324	110	*indica*
Baipigu	China	1.13	0.0494	160	*indica*
Basmati113	India	1.09	0.059	170	*indica*
Benguzhong	China	0.27	0.0305	125	*indica*
Changainuo	China	0.23	0.0317	107.4	*indica*
Dabaihangu	China	1.44	0.0476	178	*indica*
Dahongjiao-3	China	0.71	0.0129	163	*indica*
Danuo	China	0.88	0.0567	118	*indica*
Dayemaozhan	China	0.91	0.0384	92	*indica*
Erpozao	China	0.76	0.0106	93	*indica*
Hanzhangu	China	0.31	0.0069	129.6	*indica*
Hanzhangu	China	0.59	0.0189	129.6	*indica*
Hongjiaowen	China	0.4	0.0145	154.8	*indica*
IR24	Philippine	0.97	0.0591	105	*indica*
Liuyuegu	China	1.04	0.0913	87	*indica*
Maweibaimizhan	China	0.72	0.0329	179.8	*indica*
Maweixian	China	0.76	0.1124	143	*indica*
Nanjing-6	China	0.68	0.0125	151	*indica*
Nantehao	China	1.17	0.014	160	*indica*
Xiangzhou-4	China	1.26	0.0538	90	*indica*
Yangguzi-2	China	0.98	0.0977	156	*indica*
Yanzhan	China	1.78	0.0549	166	*indica*
Yanzhanzhong	China	0.78	0.0337	140.5	*indica*
Yezhugu	China	1.34	0.1526	168	*indica*
Zaobaigu	China	0.84	0.0718	162.2	*indica*
Zhefu-802	China	0.74	0.0582	80	*indica*
Zhenzhunuo	China	1.22	0.0891	133.2	*indica*
O.granulata I	China	0.027	0.0017	45.9	*granulata*
O.granulata II	China	0.03	0.0016	79.3	*granulata*
Dular	India	1.27	0.0312	165	*aus*
