# Supplementary material for: Nucleolar DEAD-Box RNA Helicase TOGR1 Regulates Thermotolerant Growth as a Pre-rRNA Chaperone in Rice
Source: PLoS Genet. 2016 Feb 5;12(2):e1005844. doi: 10.1371/journal.pgen.1005844 (PMC4743921; doi:10.1371/journal.pgen.1005844)
Supplement: S3 Table — (PDF) [file pgen.1005844.s019.pdf]

S3 Table. GO functional enrichment analysis of differently expressed genes (DEGs, ≥2-fold change) following a temperature increase in WT and mutant (*togr1*) seedlings.

GO terms	WT			*togr1*			genome	
	number of DEGs	cluster frequency	corrected P-value of enrichment	number of DEGs	cluster frequency	corrected P-value of enrichment	number of genes	genome frequency
Polysaccharide metabolic process	44	4.0%	0.00031	24	2.7%	1	310	1.8%
Carbohydrate metabolic process	88	7.9%	0.00108	56	6.3%	1	831	4.8%
Glucan metabolic process	32	2.9%	0.00399	17	1.9%	1	215	1.2%
Carbon fixation	9	0.8%	0.0055	0	-	-	24	0.1%
Organic substance metabolic process	10	0.9%	0.0456	0	-	-	37	0.2%
Response to stimulus	249	22.5%	0.03832	218	24.6%	0.00017	3124	18.0%
Response to stress	129	11.6%	1	118	13.3%	0.0078	1576	9.1%
Response to oxidative stress	5	0.5%	1	9	1.0%	0.00385	28	0.2%
Total	1108			885			17310	

All DEGs following a temperature increase from 25 ℃ to 30 ℃ were mapped to GO terms in the database (http://www.geneontology.org/) and gene numbers for every term were calculated. Ultra-geometric test was used to find significantly enriched GO terms in DEGs comparing to the genome background.
