# Supplementary material for: Nucleolar DEAD-Box RNA Helicase TOGR1 Regulates Thermotolerant Growth as a Pre-rRNA Chaperone in Rice
Source: PLoS Genet. 2016 Feb 5;12(2):e1005844. doi: 10.1371/journal.pgen.1005844 (PMC4743921; doi:10.1371/journal.pgen.1005844)
Supplement: S1 Text — (PDF) [file pgen.1005844.s020.pdf]

S1 Text. Supplemental experimental procedures

Constructs and Genetic Transformation

To construct the plasmid for *TOGR1* complementation and overexpression, a DNA fragment containing the entire open reading frame (ORF) of *TOGR1*, a 1811 bp upstream sequence, and a 1964 bp downstream sequence was prepared by digesting *BAC AC133930* with *Hin*dIII, and cloned into the binary vector *pCAMBIA1300* (Cambia). A 1792 bp upstream sequence obtained from the same BAC using *Hind*III and *Sac*I was cloned into *pCAMBIA1300* and used as control.

To construct the *35S::TOGR1:GFP*, *35S::togr1-1:GFP*, *35S::togr1-2:GFP*, *35S::togr1-3:GFP* and *35S::togr1-4:GFP* plasmids, the ORFs of *TOGR1*, *togr1-1*, *togr1-2*, *togr1-3* and *togr1-4* without the termination codon were amplified from the cDNAs of wild-type Zhongxian 3037 and the four allelic *togr1* mutants, respectively, using primers *TOGR1CF1* and *TOGR1CR1* and subcloned into the *Hin*dIII-digested binary vector *pJIT163-hGFP* (Guerineau et al., 1992). The marker construct *35S::HDT1:RFP* was generated by amplifying the CDS of HDT1 from *Arabidopsis* cDNA using primers *HDT1F* and *HDT1R* and subcloned into a pBI221-RFP vector (BioVector) at the *Bam*HI-*Hind*III site.

To construct the $TOGR1_{pro}$*::GUS* plasmid, a putative promoter of *TOGR1* was amplified from the genomic DNA of Zhongxian 3037 using primers *TOGR1PF1* and *TOGR1PR1* and subcloned into the *Pst*I-*Nco*I-digested binary vector *pCAMBIA1301*. To construct the *UBi-1::TOGR1:HA* plasmid for rice transformation, the ORF of *TOGR1* without the termination codon was amplified from the cDNAs of Zhongxian 3037 using primers *TOGR1CF2* and *TOGR1CR2* and subcloned into the *Kpn*I-*Sac*I-digested vector *pTCK303*. The *GAL1::TOGR1:HA* and *GAL1::togr1-1:HA* plasmids for yeast transformation were generated by amplifying the DNA fragments from the cDNAs of Zhongxian 3037 and *togr1-1* respectively using primers *TOGR1CF3* and *TOGR1CR2* and cloning them into the *Hin*dIII-*Sac*I-digested vector *pYES2* (Invitrogen).

The *togr1-1* mutant was transformed was transformed for complementation and immunoprecipitation analysis, a rice variety Zhonghua 11 was transformed for GUS histochemical analysis and Yandao 8 was transformed for overexpression analysis . Transgenic rice plants were generated as described previously (Hiei et al., 1994). Successfully transformed lines were further confirmed by PCR amplification of the CaMV35S promoter using primers *35SF*/*35SR*. Partial 17S rRNA gene amplified by primers *17SF*/*17SR* was used as reference.

Yeast strains used for growth assay and immunoprecipitation analysis were transformed by the lithium acetate method (Hill et al., 1991).

Tissue Stain

Internodes collected from plants grown in Beijing's summer-autumn field at heading stage, and leaf blades and roots collected from 2-week-old seedlings grown at 35 ℃ were first fixed in solution containing 10% formalin, 5% acetic acid and 50% ethanol and dehydrated in a graded ethanol series. The tissues were then embedded in paraffin and sectioned (8 μm thick). The cross-sections were floated on a drop of water on a glass slide at 42 ℃, and dried at the same temperature overnight. After being deparaffinized, the cross-sections were stained by 2% (w/v) safranin water solution and 2% (w/v) fast green in 95% ethanol and sealed by Canada balsam.

Leaf sheaths collected from 20-day old seedlings were first fixed in a solution containing 5% formalin, 5% acetic acid and 60% ethanol and then dehydrated in a graded ethanol series. Fixed tissues were cut into 3-4 mm squared pieces, stained by 0.005% (w/v) safranin for two days, and subsequently mounted on glass slides.

The slides were observed and photographed under a light microscope (BX51; Olympus).

Positional Cloning

The F2 population for map-based cloning was generated from a cross between *togr1-1* and a *japonica* variety Zhonghua-11. The rice plants were grown in the paddy fields under Beijing's hot summer conditions. Genomic DNA was extracted from leaves using the CTAB method. The primers (Table S1) for the sequence tagged site (STS) and derived cleaved amplified polymorphic sequences (dCAPS) markers (Table S1)

developed on the basis of the sequence polymorphisms between *japonica* and *indica* (http://www.gramene.org/resources/) and rTaq (Takara Bio) were used for amplification. PCR products were separated on 4.0% agarose gels, and linkage analysis was performed between the *togr1-1* mutation and markers.

List of the PCR-based molecular markers developed for positional cloning

Marker[a]	Primer pairs
P1	F 5′-ACGCTAGAGACTAATGGCTT-3′
	R 5′-CTGCTCAGGCATTAAAAAGG-3′
P2	F 5′-CCCTCTGTATCTGTTTCATA-3′
	R 5′-CACTAACTACGACACCAAAT-3′
P3	F 5′-ACAACTACATTGCGACTGTA-3′
	R 5′-TAGCACTATACTTTCTCCGT-3′
P4	F 5′-CGGTTGTCTTTCATTTTCTC-3′
	R 5′-CAATTCTGCAAAATGAAGCA-3′
P5	F 5′-CATGGAGACAATAGATGAGA-3′
	R 5′-ACAGGCCCATAGAAGTCTTT-3′
P6	F 5′-TCAGTGAACCAGCCACATGA-3′
	R 5′-CTTTCGCGGTTCTTGGAGCA-3′
P7	F 5′-CTAGCGAGTGAGAGTTATG-3′
	R 5′-GTAAGGTCTTGGTTGTTAGG-3′
P8	F 5′-AGGACGAGAAAAGTGAAGAGA-3′
	R 5′-CAACAACTGACGATGAATCTA-3′
P9	F 5′-CTGATCTTTGCGTTAATGATG -3′
	R 5′-GAGATCCTAGGCACATATAT-3′
P10	F 5′-TGCTTGTGCACATGTTTAGT-3′
	R 5′-TAATCACCATGGCAAACAAC-3′

[a]The markers are sequence tagged site (STS) and derived cleaved amplified polymorphic sequences (dCAPS) markers.

Subcellular Localization of the GFP Fusion Proteins

The fusion constructs (*35S::TOGR1:GFP, 35S::togr1-1:GFP, 35S::togr1-2:GFP, 35S::togr1-3:GFP* and *35S::togr1-4:GFP*) and control (*35S::GFP*) were transiently expressed in rice protoplast as described (Bart et al., 2006). Briefly, the lower halves of 10-day old etiolated rice seedlings grown in the dark at 28 ℃ were first cut into small pieces in 0.6 M mannitol and digested in an enzyme solution (0.6 M mannitol, 10mM MES (pH5.7), 1.5% (W/V) cellulase RS (Yakult), 0.75% (W/V) macerozyme (Yakult), 0.1% BSA (W/V), 1mM $Cacl_2$, 5mM β-mercaptoethanol and 50 μg/ml carbenicillin) as being shook at 80 rpm and 28 ℃ in the dark for 5 h. Protoplasts were then washed by W5 solution (154mM NaCl, 125mM CaCl2, 5mM KCl and 2mM

MES (pH5.7)) and ice cold Mmg solution (0.6M mannitol, 15 mM MgCl2 and 4mM MES (pH5.7)) and kept in 100 µl of Mmg solution for 30 min ice bath. After adding 20 µl of plasmids (1µg/µl), 120 µl of 40% PEG and 10 ml of W5, protoplasts were incubated in the dark at 28 ℃ for 24 h. Protoplasts were stained by Hoechst 33258 (Sigma-Aldrich) before being visualized and photographed under a fluorescence microscope (Olympus BX53).

Protein Expression and Purification

The ORFs of *TOGR1* and *togr1-1*were amplified from the cDNAs of wild-type Zhongxian 3037 and the *togr1-1* mutant, respectively, using primers *TOGR1CF5* and *TOGR1CR5* and were inserted into a vector between the BamHI and XhoI sites. The vector was engineered from pET-30a (Novagen) with a SUMO tag and a Prescission protease recognition sequence at its C-terminal cloned into it between the NcoI and BamHI sites. His_6-SUMO-tagged fusion proteins were then expressed in an *E. coli* strain Transetta (TransGen Biotech) at 16 ℃ and cell lysates were prepared by sonication. The desired proteins were purified using Ni–NTA (Novagen) and eluted with buffer containing 25 mM Tris–HCl (pH 8.0), 150 mM NaCl, 250 mM imidazole. His_6-sumo was cleaved from the purified proteins by GST-tagged Prescission protease (GE Healthcare) at a ratio of 1:100 (protease to target protein; w/w), and His_6-sumo residue and protease were subsequently removed by Ni-NTA resin (Invitrogen) and Glutathione Sepharose 4B (GE Healthcare) respectively.

Helicase Assay

RNA substrates were synthesized using solid-phase phosphoramidite chemistry, purified by HPLC and labeled at the 5′ end of the indicated strand with ROX fluorophore (Takara Bio). Helicase assay was modified from a method previously described (Turner et al., 2007). Briefly, 20 µl of reaction mixtures containing 10 µM purified protein, 20 mM Tris-HCl (pH 7.6), 5 mM $MgCl_2$, 2 mM ATP, 0.1 mg/ml bovine serum albumin, 1 µM ROX-labeled RNA duplex and 20 µM trap RNA were incubated under specified conditions. Aliquots (10 µl) were then quenched in 5 µl of stop solution containing 10 mM EDTA, 40% glycerol, 0.025% bromophenol blue and 0.025% xylene cyanol and kept on ice before running on a 17% native polyacylamide

gel in 1×TBE buffer. The gel was then sealed with transparency and imaged on Typhoon Trio imager (GE Healthcare) at 532 nm excitation wavelength and 630±30 nm emission wavelength.

Yeast Growth Assays

For complementation analysis, YPH499 (*MAT*a *ura3-52 lys2-80 ade2-101 trp1-Δ63 his3-Δ200 leu2-Δ1*) in which the chromosomally encoded *Rrp3* was placed under the control of the DOX-repressible *tetO7* promoter was used as parent strain (Granneman et al., 2006). Strain carrying empty *pYES2* vector or *pYES2* plasmid containing *GAL1::TOGR1:HA* or *GAL1::togr1-1:HA* was grown in synthetic minimal medium (6.7 mg/ml YNB, 0.8 mg/ml –URA DO supplement (Clontech), 2% dextrose) to exponential phase and then washed with water. Three 10-fold serial dilutions at OD600 0.5, 0.01 and 0.002 were made in water and 10 μl of each dilution was spotted on induction medium (6.7 mg/ml YNB, 0.8 mg/ml –URA DO supplement, 2% agar, 2% D-galactose, and 2% D-raffinose) supplemented with 2 μg/ml DOX. Growth curves were monitored in liquid induction medium supplemented with 60 μg/ml DOX with starting OD600 = 0.015, and strain carrying empty *pYES2* grown in liquid synthetic minimal medium was used as control.

Immunoprecipitation

For rice, *UBi-1::TOGR1:HA* plants in *togr1-1* background were grown for 60 days in growth chamber. Wild-type Zhongxian 3037 and *UBi-1::OsPRR1:HA* plants in Zhonghua-11 background were used as negative controls. Leaf blades were harvest and plant whole-cell extracts were prepared as previously described (Dunoyer et al., 2007).

For yeast, *tetO7::Rrp3* YPH499 strain harbouring *GAL1::TOGR1:HA* or *GAL1::togr1-1:HA* were cultured in induction medium containing D-galactose and DOX at 30 ℃. The *GAL1::TOGR1:HA* strain and the parent strain grown in the non-induction SC-U medium were used as negative controls. At exponential stage, yeast whole-cell extracts were prepared according to a published method (Lee and Baserga, 1999) using glass beads (Sigma-Aldrich).

Immunoprecipitation was performed as previously described (Dunbar et al., 1997). Total RNA was then extracted for northern analysis.

RNA Methods

Total RNA was extracted with the TRIzol reagent (Invitrogen) according to the manufacturer's instructions.

For northern blotting, 5 μg of total RNA was separated on 1.2% agarose gels containing 6% formaldehyde and then blotted onto Hybond N+ membranes (GE healthcare) by capillary elution. For hybridization, RNA probes antisense to the rice or yeast U3 snoRNA labeled with [α-^{32}P] UTP using T7 RNA polymerase (Takara Bio) and oligonucleotides labeled with [γ-^{32}P]ATP at the 5′ ends using T4 polynucleotide kinase (Promega) were used to detect the U3 snoRNAs and pre-rRNA processing intermediates, respectively. Membranes were hybridized in PerfectHyb Plus hybridization buffer (Sigma-Aldrich) according to the manufacturer's instructions and analyzed by exposure to X-ray films.

The 5′ and 3′ ends of rRNA precursors were determined by circular RT-PCR as described previously (Perrin et al., 2004).

For qRT-PCR, total RNA was first digested with RNase-free DNaseI (Takara Bio). Oligo (dT) primer (*CDSIII*) and M-MLV reverse transcriptase (Invitrogen) were used to produce complementary cDNA from 1 mg of RNA following the manufacturer's instructions. Quantitative PCR was performed on a CFX96 real-time PCR detection system (Bio-Rad) using Power SYBR green PCR master mix (Applied Biosystems). Relative transcript levels were determined using the comparative threshold cycle (Ct) method. Primer pairs *TOGR1QF1*/ *TOGR1QR1*, *Q1F*/*Q1R*, *Q2F*/*Q2R*, *Q3F*/*Q3R*, *OsACTIN1F*/ *OsACTIN1R* and *OsTUB1F/OsTUB1R* were used to detect transcripts of *TOGR1*, *LOC_Os07g49400*, *LOC_Os03g20120*, *LOC_Os07g48830* and normalization controls *Actin1* and *α-Tubulin*, respectively. Three biological and three technical repeats were performed in the experiments.

Transcriptome Resequencing and Gene Ontology (GO) Analysis

Four-day-old Zhongxian 3037 and *togr1-1* seedlings were both divided into two groups and grown in growth chambers at 25 ℃ and 30 ℃ respectively for further 18

days. For each treatment, leaf blades of the fourth leaves were collected, pooled into 100 mg and frozen in liquid nitrogen. Four samples in total were obtained, including Zhongxian 3037 at 25 ℃ and 30 ℃ and *togr1-1* at 25 ℃ and 30 ℃. RNA sequencing was conducted by BGI (Shenzhen, China) using total RNA extracted with the TRIzol reagent. Gene expression level was calculated using RPKM (reads per kb per million reads) method.

Protein Gel and Blot Analysis

Plant leaves or yeast cells were frozen in liquid nitrogen, ground into powder and treated with sodium phosphate buffer (pH 7.4) containing 50 mM β-glycerophosphate, 100 μM Na_3VO_4, 0.5 mM phenylmethanesulfonylfluoride (PMSF), and Complete Protease Inhibitor Cocktail (Roche) for crude protein isolation. Protein samples were loaded and separated on SDS-PAGE gels. For direct visualization, they were stained with Coomassie brilliant blue. For blot analysis, they were transferred to Immobilon -P PVDF (Polyvinylidene Difluoride) membrane (Merck Millipore). Mouse monoclonal anti- HA (Sigma-Aldrich) and goat anti-mouse IgG horseradish peroxidase conjugate (Sigma-Aldrich) were used as primary and secondary antibodies, respectively. OsACTIN1 was analyzed as an internal control. After being incubated with Immobilon Western Chemiluminescent HRP substrate (Merck Millipore) and exposed to X-film, proteins were visualized and photographed.

Primers used in this study

Name	sequence	Note
TOGR1T1F	5′-GCCTCCTCCTTCCTTCTTTAACCG-3′	TILLING
TOGR1T1R	5′-TAACAACATTCATAAAACACCAATCG-3′	
TOGR1T2F	5′-GCCAAATGAGTCAGGTTAGTATGCTT-3′	TILLING
TOGR1T2R	5′-AATTCGTCGAAATGGACCTACATAAA-3′	
TOGR1CF1	5′-AAGCTTATGGCGAAGAAGAAGGA-3′	Cloning
TOGR1CR1	5′-AAGCTTCCGTCTTCTTGATTTGTTG-3′	
TOGR1CF2	5′-GGTACCATGGCGAAGAAGAAGGACGT-3′	Cloning
TOGR1CR2	5′-GAGCTCTCAAGCGTAATCTGGAACATCGTAT GGGTAAGCCCGTCTTCTTGATTTGTTGAAG-3′	
TOGR1CF3	5′-AAGCTTATGGCGAAGAAGAAGGACGT-3′	Cloning
TOGR1CR3	5′-GAGCTCTCACCGTCTTCTTGATTTGTTG-3′	

TOGR1CF4	5′-TCTAGAATGGCGAAGAAGAAGGACGT-3	Cloning
TOGR1CR4	5′-AAGCTTCCGTCTTCTTGATTTGTTG-3′	
TOGR1CF5	5′-GAAATTAGATCTATGGCGAAGAAGAAGGACGT-3′	Cloning
TOGR1CR5	5′-GAAATTCTCGAGTCACCGTCTTCTTGATTTGTTG-3′	
TOGR1PF1	5′-CTGCAGCCACCCGAGGGGTAGTCTAC-3′	Cloning
TOGR1PR1	5′-CCATGGGGCTACGGAGAGACGGGGGA-3′	
TOGR1QF1	5′-AGGACGTGGAGGTGGAGGAGTTGG-3′	qRT-PCR for *TOGR1*
TOGR1QR1	5′-CTTCGAGGGCGTGGGGGATGG-3′	
OsACTIN1F	5′-CTTCATAGGAATGGAAGCTGCGGGTA-3′	qRT-PCR reference
OsACTIN1R	5′-CGACCACCTTGATCTTCATGCTGCTA-3′	
OsTUBULINF	5′-GGAAATACATGGCTTGCTGCTT-3′	qRT-PCR reference
OsTUBULINR	5′-TCTCTTCGTCTTGATCGTTGCA-3′	
U3RT7F	5′-AGATAATACGACTCACTATAGGGCCTGTCAG ACAACCTGAGA-3′	Rice U3 snoRNA probe preparation
U3RT7R	5′-ACGACCTTACTTGAACAGGATC-3′	
U3YT7F	5′-AGATAATACGACTCACTATAGGGACTTGTCA GACTGCCATTTGTA-3′	Yeast U3 snoRNA probe preparation
U3YT7R	5′-CTATAGGAATCGTCACTCTTTG-3′	
35SF	5′-AGAGATAGATTTGTAGAGAGAG-3′	Transgene confirmation
35SR	5′-ATGGTGGAGCACGACACTC-3′	
17SF	5′-CGGCTACCACATCCAAGGAA-3′	PCR reference
17SR	5′-TGTCACTACCTCCCCGTGTCA-3′	
HDT1F	5′-GCCGGATCCATGGAGTTCTGGGGAATTG-3′	Cloning
HDT1R	5′-GCCAAGCTTCTTGGCAGCAGCGTGC-3′	
Q1F	5′-AGAGTCAGTACGATCAAGAC-3′	qRT-PCR for *LOC_Os07g49400*
Q1R	5′-TCTTGACAGCAAATAGCTTGG-3′	
Q2F	5′-ACGTATGCTGTGGATTTTGTGA-3′	qRT-PCR for *LOC_Os03g20120*
Q2R	5′-GGCCGTAATTTTTGCAACTTCC-3′	
Q3F	5′-CATGTACGTTACTGCTGATACATT-3′	qRT-PCR for *LOC_Os07g48830*
Q3R	5′-TAACAACACTGAGAAAGACATTGAC-3′	

Supplemental References

Guerineau, F., Lucy, A., and Mullineaux, P. (1992) Effect of two consensus sequences preceding the translation initiator codon on gene expression in plant protoplasts. Plant Mol. Biol. *18*, 815-818.

Hiei, Y., Ohta, S., Komari, T., and Kumashiro, T. (1994). Efficient transformation of rice (*Oryza sativa* L.) mediated by *Agrobacterium* and sequence analysis of the boundaries of the T-DNA. Plant J. *6*, 271-282.

Hill, J., Donald, K., and Griffiths, D. E. (1991). DMSO-enhanced whole cell yeast transformation. Nucleic Acids Res. *19*, 5791-5791.
